# Recycling Poly(Butylene Succinate)/Brewer’s Spent Grain Pot Prototypes

**DOI:** 10.3390/polym18172119

**Published:** 2026-08-31

**Authors:** Annamaria Visco, Gianmarco Sindona, Cristina Scolaro, Salim Brahimi

**Affiliations:** Department of Engineering, University of Messina, C.da Di Dio, 98166 Messina, Italy; cscolaro@unime.it (C.S.); salim.brahimi@unime.it (S.B.)

**Keywords:** recycled bioplastic, agri-food waste, processability, mechanical performance, thermal stability

## Abstract

The recyclability of pots made entirely from brewer’s grains (BSGs) and poly(butylene succinate) (PBS) at a PBS/BSG weight ratio of 70:30, hereafter referred to as MIXr, was evaluated. The resulting pots exhibited tensile strength at break comparable to that of conventional fossil-based polymeric materials. The study aimed to determine the maximum content of recycled materials that could be incorporated into virgin (PBSv) while maintaining acceptable properties, thereby extending the material’s life cycle through mechanical recycling before disposal via biodegradation or composting. For comparison, blends of virgin PBS with recycled PBS (PBSv–PBSr) and recycled BSG/PBS material (PBSv–MIXr) were prepared at the same recycled-material contents. The materials were characterized in terms of processability, tensile properties, thermal stability (TGA), chemical group analysis (FTIR), and surface properties. The results showed that the presence of BSG significantly influences the recyclability of the biocomposite. While virgin PBS could incorporate up to 10 wt.% recycled PBS without a significant loss in ductility or thermal stability, PBSv–MIXr blends exhibit a ~20–30% reduction in deformability at MIXr contents ranging from 2 wt.% to 20 wt.% and a reduction in thermal stability ranging from 16–22% at levels of incorporation ranging between 10 wt.% and 40 wt.%.

## 1. Introduction

This study aims to provide new insights into the recyclability of produced prototypes (pots) made from a blend of biodegradable/bioderived bioplastics and industrial agri-food waste, while also evaluating the role of the agri-food waste in the recycling process.

The growing environmental concerns associated with conventional fossil-based plastics have stimulated the development of more sustainable polymeric materials, particularly bio-based, biodegradable, and compostable bioplastics [1,2,3].

Despite their promising potential to reduce plastic waste accumulation, further research is still needed to assess their overall suitability as alternatives to conventional plastics in terms of performance, functionality, and long-term environmental impact [4,5,6]. Moreover, many biodegradable polymers degrade efficiently only under controlled industrial composting conditions, whereas their persistence in natural environments may still raise environmental concerns [6]. Although industrial composting is currently one of the most widely adopted end-of-life strategies for these materials, it results in complete material degradation; therefore, alternative approaches aimed at extending material utility through recovery and reutilization should be further explored [4,5]. Although they still account for a relatively small share of the polymer market, bio-based and biodegradable plastics are gaining increasing commercial importance. This group includes several widely studied materials, such as polylactic acid (PLA), polyhydroxyalkanoates (PHA), polyhydroxybutyrate (PHB), poly(butylene adipate-co-terephthalate) (PBAT), poly(butylene succinate) (PBS), polycaprolactone (PCL), and thermoplastic starch (TPS). Their widespread use in food packaging and disposable products has made industrial composting, performed under controlled temperature and humidity, one of the most commonly adopted end-of-life (EoL) strategies [7].

The principal EoL routes for PBS-based products after their first use (namely landfilling, biodegradation/composting, and incineration) ultimately lead to the complete degradation or destruction of the material [4,8]. Therefore, before pursuing these terminal options, it is important to consider strategies to extend the functional value of bioplastics beyond their initial use phase [5].

Mechanical recycling represents a valuable strategy for extending the service life of post-consumer bioplastics [9]. According to Garofalo et al., the mechanical recycling of materials such as PLA and PBS may provide greater environmental benefits than landfilling or industrial composting, primarily because it results in lower net CO_2_ emissions [10]. Unlike terminal end-of-life (EoL) options, recycling enables the regeneration and reuse of polymers for the same or secondary applications, thereby avoiding the environmental impacts associated with the production of virgin materials. The same authors also highlighted the industrial relevance of this approach, as it can both reduce production costs and improve the sustainability of polymer-processing value chains. This is especially important in high-throughput manufacturing technologies for single-use products (including extrusion, blow molding, thermoforming, and injection molding), which inherently generate substantial amounts of recyclable process scrap. As this practice is already well-established in fossil-based polymer systems, it could also be applied to bioplastics, provided that material properties are adequately retained [11,12,13,14]. Overall, the use of bioplastics for extended life cycles requires understanding and controlling chemical, structural, and morphological changes during recycling, linking material properties to potential secondary applications [11].

In this context, pellets of PBS can generally withstand up to approximately four to five reprocessing cycles before the reduction in molar mass compromises their performance [12]. These results confirm previous studies on PBS recycling, which highlight good mechanical properties, supporting its use in closed-loop or cascade recycling processes [13,14]. However, the mechanical recycling of PBS is primarily limited by the reduction in molecular mass due to hydrolysis during thermal treatment in the presence of moisture. Furthermore, PBS is subject to physical aging, with rearrangements in the amorphous domains that can reduce chain mobility and alter its mechanical and rheological properties over time [13]. Among strategies for limiting molecular mass loss, vitrimerization is an upcycling approach that transforms the polymer into a dynamic network that is stable yet capable of reorganizing upon heating, thereby enabling multiple reprocessing cycles and greater degradation resistance [14].

**Figure 1:**
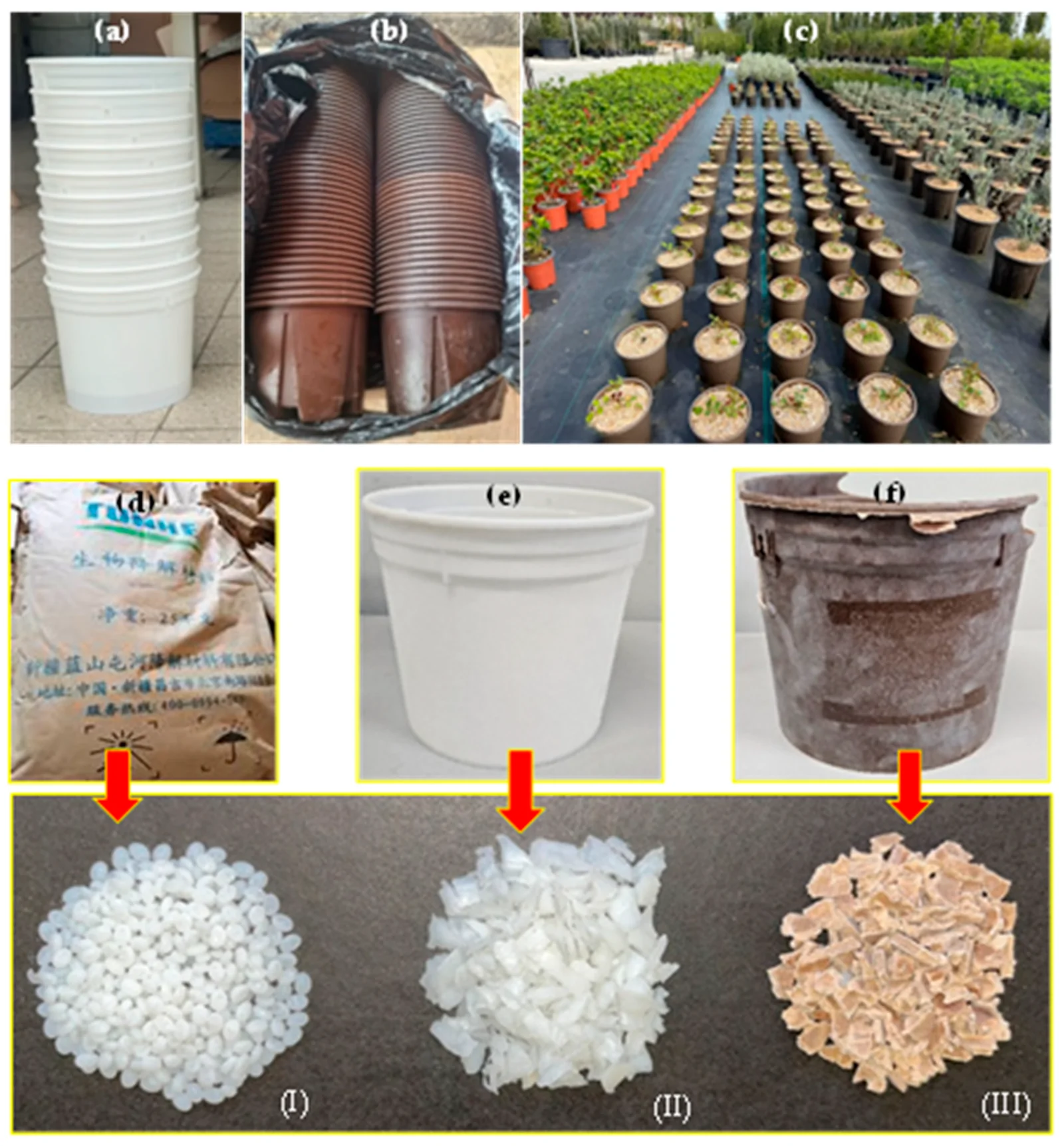
White commercial pots made from virgin PBS and used as reference material (**a**); brown pots made from PBS containing 30 wt.% BSG (**b**); brown pots in a nursery (**c**). Materials: PBSv pellets (**I**), provided by Tunhe Sci. & Tech. Co. [19] (**d**); PBSr flakes (**II**), obtained by crushing pots made from PBS (**e**); MIXr flakes (**III**), obtained by crushing pots made from PBS/BSG-70/30 (wt./wt.) (**f**).

In the present study, we reused commercial pot prototypes produced by injection molding. The use of natural agri-food waste in both bio-derived and biodegradable bioplastics such as PBS makes the resulting bio-composites a material. Two types of pots were investigated: white-color pots made of pure PBS, and brown-color pots (made of 70/30 wt.% PBS/BSG bio-composite in which PBS is mixed with 30 wt.% industrial agri-food waste from the brewery called Brewer’s Spent Grain (BSG); see Figure 1). The use of agri-food waste in bio-composites represents a strategy for the valorization of agro-industrial residues produced in Sicily, as already highlighted in our previous studies [15,16]. Both pot types were ground and remixed with virgin PBS granules to produce blends of virgin material (v) mixed with recycled material (r) at concentrations ranging from 2 to 40 wt.%. All the starting materials (i.e., PBS, BSG, and 70/30% PBS/BSG) have been previously studied by our group to evaluate chemical, physical, and mechanical biodegradability and processing aspects [12,16,17].

While our previous studies investigated the aging and recycling behavior of PBS-based biodegradable pots, the present work is the first to systematically evaluate the effect of increasing contents of post-warehouse recycled pot regrind incorporated into virgin PBS. In this study, we demonstrated that bio-composite pots also have acceptable mechanical performance compared to traditional pots suitable for horticultural applications, made with fossil-based polymers (polyolefins), and we paid particular attention to the role that BSG plays in pot recycling. To the best of our knowledge, there are no further comparative data or systematic studies to assess the maximum amount of this recycled biocomposite to be added to PBSv without compromising its physical–mechanical performance. The findings of this study are particularly relevant to scenarios in which commercial containers are recovered for recycling after a long period of use and/or warehouse storage, as is often the case in industrial practice. This aspect is of particular significance given that the European market for plant pots is estimated at around 191 million units in 2026 (increasing to 239 million units by 2031), [18].

## 2. Materials and Methods

### 2.1. Materials

The starting raw materials used in this study were as follows:

(a) Virgin PBS (code: PBSv): bioplastic supplied by Xinjiang Blue Ridge Tunhe Sci.&Tech. Co., Ltd. (No. 316 Beijing Nan Lu, Changji city, Xinjiang, China). PBS lot number: 202205272506A2, MFI = 25–30 g/10 min.
(b) Recycled PBS (code: PBSr) obtained from the crushing of prototypes of pots made from virgin PBS and prepared for injection molding by Mavi S.a.s. of Magistro Francesco, Via Ferrara 98061 Brolo, ME, Italy. The MFI of PBSr is 182.75 g/10 min.
(c) Recycled blend (code: MIXr), obtained from the crushing of pots made from pellets consisting of virgin PBS blended with brewer’s spent grain (BSG) as agrifood waste (bio-composite PBS/BSG 70/30, wt./wt.), provided by Mavi S.a.s. of Magistro Francesco, Via Ferrara 98061 Brolo, ME, Italy. No additives have been added to the pots. In detail, BSG was re-used as agrifood waste filler in a mesh < 100 μm and at 30 wt.% according to Visco et al. [16] to produce pellets of PBS/BSG 70/30 wt./wt by EcoBuddy™ bioplastics (Contrada Internicola, Zona Campo Sportivo SNC 98040 Roccavaldina, ME, Italy). Pellets were then used to produce the pots using an injection molding machine operated by Mavi S.a.s. di Magistro Francesco, Via Ferrara 98061 Brolo, ME, Italy. The discarded pots were thoroughly cleaned to remove any residual contaminants before being chopped and tested.

All materials were dried in an oven at 60 °C for 4 h to reach a humidity level of 0.08 wt.%. The raw materials used are summarized in Table 1 and illustrated in Figure 1. The melt flow rate (MFR) of virgin PBS (37.36 g/10 min) is higher than that reported in the PBS datasheet (25–30 g/10 min) [20]. This indicates the beginning of degradation of the virgin PBS, since the production date was May 2022, while the tests were performed in November 2025 on pellets taken from a still-sealed package as part of this experimental campaign.

The MFR values of the recycled materials (PBSr and MIXr) were both extremely high (159 g/10 min and 153 g/10 min, respectively), in accordance with the literature data [12]. In fact, plant pots produced in 2024 and kept on shelves (warehouse storage) up to the testing date (November 2025, about one year and nine months after production) were evidently subject to environmental degradation, leading to higher flowability. The increase in melt flow index (MFI) is widely considered an indicator of polymer degradation, since chain scission reduces molecular weight and melt viscosity, thereby increasing the material’s flowability [21,22].

In this study, we analyzed pots that already showed some degree of degradation due to storage, likely attributable to hydrolysis, photodegradation, and physical aging. This initial condition is confirmed by the increase in the melt flow rate (MFR) reported in Table 1 and the reduction in mixing torque reported in Table 2. This pre-existing degradation was subsequently compounded by that induced by mechanical recycling, which may have caused polymer chain scission and/or cross-linking during reprocessing. Since all the reprocessed materials came from artifacts subjected to the same storage conditions and, therefore, characterized by a similar initial state of degradation, the analysis focused on the effect of reprocessing alone, comparing the samples starting from the same initial degradation condition.

Table 1 lists the melt density obtained during MFR measurements (see Section 2.2.1 below). The higher density value of MIXr (1.20 g/cm^3^) compared to PBSr (0.99 g/cm^3^) is due to the presence of BSG in the amount of 30 wt.%. The theoretical density of BSG is 1.404 g/cm^3^ and is higher than the theoretical density of PBS (1.260 g/cm^3^), as already analyzed and discussed in detail in our previous work [20].

To perform the characterization tests, biocomposite sheets were prepared by melt compounding the raw materials listed in Table 2 in a Brabender Plasticorder PL2100 at 140 °C for 10 min and 40 rpm. The selected processing conditions were based on previous studies on PBS-based composites [17,23] and were considered suitable to ensure effective melting and mixing while minimizing the risk of thermal degradation, which is reported to become significant above 190 °C [24].

Table 2 reports the composition of all the blends and the actual BSG content in the PBSv–MIXr formulations, which decreases from the initial 30 wt.% in MIXr due to dilution to lower values ranging from 0.6 wt.% (in PBSv–MIXr2) to 12 wt.% (in PBSv–MIXr40). In the same Table 2, the amount of PBS in each PBSv–MIXr blend is detailed. The resulting blends were then compression-molded into 12 cm × 12 cm sheets with a thickness of 1 mm using a hydraulic press and a three-stage cycle: 7 min at 0 bar, 5 min at 50 bar, and 3 min at 100 bar.

**Figure 2:**
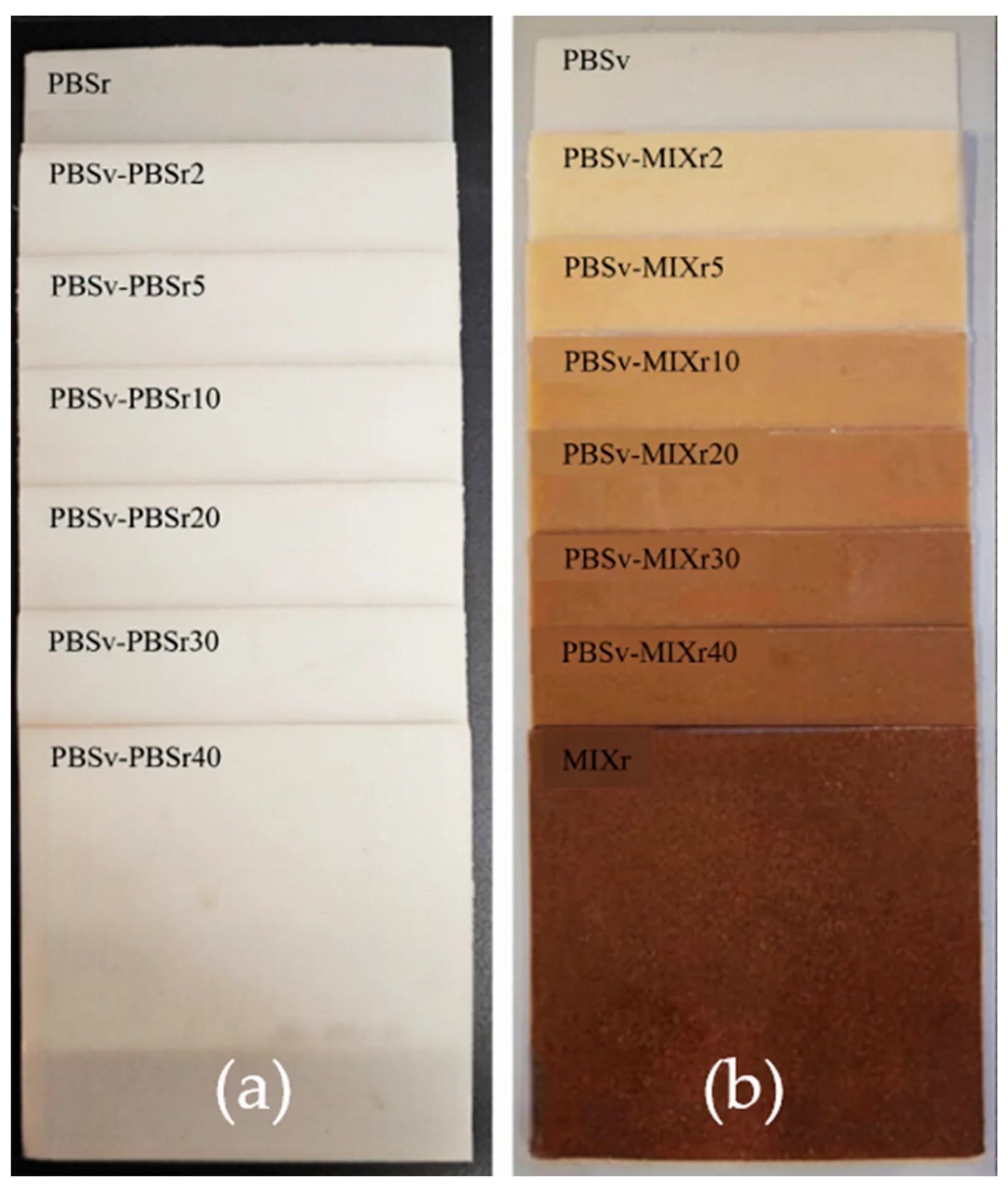
Photographs of PBSv-PBSr0–2–5–10–20–30–40 (**a**) and of PBSv-MIXr0–2–5–10–20–30–40 (**b**) sheets.

The produced sheets are shown in Figure 2a,b; they appear generally uniform. The sheets were superimposed to better highlight the color variation. The color of virgin PBS is typically white, while the color of MIX is dark brown. The color of PBSv-PBSr remains white regardless of the PBSr amount (Figure 2a). The color of the PBSv-MIXr blends is intermediate between white and dark brown, assuming a progressive darkening with increasing MIXr content (different shades of light brown/beige; Figure 2b).

### 2.2. Characterization Analyses

Virgin PBS was pre-dried at 60 °C for 4 h to reach a humidity level (HL) of 0.08%, PBSr was pre-dried at 60 °C for 4 h to reach an HL value of 0.06%, and MIXr was also pre-dried at 60 °C/overnight to reach an HL of 0.12%. To keep HL as low as possible, we consider the following threshold values that should not be exceeded: 0.08 wt.% HL for PBS and 2 wt.% HL regarding BSG in the MIXr bio-composite. The humidity level of all the materials has been checked by an AXIS moisture analyzer, mod. AST120 provided by AXIS Sp. z o.o. 80–125 Gdańsk, Poland, by heating the samples at 60 °C.

#### 2.2.1. Melt Flow Rate (MFR)

The MFR of the bio composites was measured using an Instron melt flow indexer (model C-MFI5, system ID MFI5-B31599) supplied by ITW Test Measurement Italia S.r.l., Pianezza, Torino, Italy. Measurements were performed in accordance with ISO 1133 [25] under a load of 2.16 kg at 190 °C, following the B/C test procedure. Each sample was tested in triplicate, and the mean value was used for analysis. The test allowed us to know the MFR and the melt density values, both listed in Table 1.

#### 2.2.2. Torque Analysis

During the melt-compounding process, processability was evaluated using mixing torque measurements. Torque values were continuously recorded during the mixing using WINMIX 4.9.1 software, on a Brabender Plasticorder PL2100 system (Belotti Instruments, Peschiera Borromeo, Milan, Italy), to assess the mixing behavior of all raw materials and blends listed in Table 2. The reported torque–time curves represent the average of three independent experimental runs.

#### 2.2.3. Mechanical Characterization

To evaluate tensile behavior, all sheets were cut using Ray-Ran Test Equipment Ltd. (Model: RR/HCP/124, Nuneaton, UK) to obtain dog-bone specimens. The specimen geometry was in accordance with ASTM D638 [26], with a width of 2.5 mm, a thickness of 1 mm, and a gauge length of 15 mm. Tensile properties were evaluated using a Lloyd LR10K universal testing machine (Elis—Electronic Instruments & Systems S.r.l., Rome, Italy). The tests were performed under quasi-static conditions with a 0.5 kN load cell, applying an initial preload of 1.00 N and a crosshead speed of 2 mm/min. All measurements were carried out under laboratory conditions (approximately 20 °C and 65% relative humidity) in accordance with the ASTM D638 standard. The mechanical parameters derived from the stress–strain curves and discussed in this study include Young’s modulus (E), tensile strength (σ_r_), elongation at break (ε_r_), and work at break (W). Eight specimens were tested for each formulation to minimize the impact of individual inconsistencies or measurement errors. It should be noted that mechanical tests performed on sheets obtained through simple thermoforming allow us to characterize the mechanical behavior of the material processed with this technology. However, the mechanical properties may differ from those of the material obtained through injection molding, the technology currently used for vase production. This is because, during the two different transformation processes, the molten polymer is subjected to different flow conditions, strain rates, pressure, and cooling, which can influence the microstructure and, consequently, the mechanical behavior of the final product.

#### 2.2.4. Thermogravimetric Analysis

Thermogravimetric analysis (TGA) was conducted using TA Instruments SDT Q600 (New Castle, DE, USA) under an argon flow of 100 mL/min to determine the thermal decomposition temperatures of each sample. Approximately 10 mg of each selected sample was analyzed. Samples were initially heated from room temperature to 105 °C at a rate of 10 °C/min, followed by an isothermal hold at 105 °C for 10 min to eliminate residual moisture. Subsequently, the samples were heated at 10 °C/min up to 700 °C, then were cooled to room temperature under laboratory ambient atmosphere. TGA profiles were evaluated in accordance with ASTM E2550 [27]. The primary parameters considered included: T_onset_, defined as the point on the thermogravimetric curve where the first deviation from the baseline occurs before the thermal degradation event; T_max_, the temperature corresponding to the maximum rate of mass change; and Δ_w_, representing the change in sample mass as a function of temperature or time. Experimentally, Δ_w_ was determined from the integration of the corresponding mass loss across the baseline boundary of the derivative thermogravimetric (DTG) peak, expressed as a percentage.

#### 2.2.5. Fourier Transform Infrared Spectroscopy (FTIR)

FTIR Analysis: IR spectra were recorded using a “Spectrum Two” Perkin–Elmer FT-IR spectrometer (Shelton, CT 06484, USA) equipped with an attenuated total reflectance (ATR) accessory. Spectra were acquired over a wavenumber range of 4000–500 cm^−1^, at a spectral resolution of 4 cm^−1^ and 32 scans per sample. Background correction was performed before each acquisition. Spectra were processed using Spectrum 10 as IR software and Origin 2018 version 9.5 by OriginLab Corporation. Samples were dried for 4 h at 60 °C before testing.

The formation of oxidate species (carbonyl and hydroxyl) indices were calculated using Equations (1) and (2), where *A* denotes the area of the corresponding peak:

(1) $$\;Carbonyl\;Index\;\left(C.I\right)=\frac{A_{1710}}{A_{2920}}$$

(2) $$\;Hydroxyl\;Index\;\left(H.I\right)=\frac{A_{3600-3200}}{A_{2920}}$$

Based on previous studies [12], the band at 2920 cm^−1^ was selected as the reference, as it shows minimal variation during the degradation process.

#### 2.2.6. Statistical Analysis

Statistical Analysis: Mechanical properties are reported as mean ± standard deviation (SD), calculated from eight independent tensile specimens for each formulation. Before inferential analyses, the assumptions required for parametric statistics were systematically verified. Residual normality was assessed using the Shapiro–Wilk, Anderson–Darling, D’Agostino–Pearson omnibus and Kolmogorov–Smirnov tests, while homogeneity of variances was evaluated using the Brown–Forsythe test. When variance homogeneity was not satisfied, Welch’s ANOVA was additionally performed to verify the robustness of the statistical conclusions.

The effects of material formulation (PBS and MIX) and recycled-content level were initially evaluated by two-way analysis of variance (two-way ANOVA), followed by Bonferroni-adjusted multiple comparisons. Although recycled content is intrinsically a quantitative variable, in the present study it was investigated as predefined experimental formulations (2, 5, 10, 20, 30, and 40 wt.%), each independently produced and mechanically characterized. Consequently, recycled content was treated as a fixed experimental factor, allowing direct comparison among the manufactured formulations and evaluation of the interaction between material formulation and recycled-content level.

To complement the factorial analysis, recycled content was also analyzed as a continuous predictor by regression analysis. Linear and, where appropriate, non-linear regression models were fitted to the experimental data, and the most appropriate model was selected according to the goodness-of-fit statistics. Regression equations, coefficients of determination (R^2^) and residual analyses were used to describe the continuous evolution of the investigated mechanical properties with increasing recycled content.

Statistical significance was established at *p* < 0.05. All statistical analyses were performed using GraphPad Prism version 8.0.2 (GraphPad Software, Inc., La Jolla, CA, USA).

#### 2.2.7. Surface Roughness

The surface roughness [28] measurements were performed using a portable and compact Surftest SJ-210 Series 178 Roughness tester (Mitutoyo S.r.l., Milan, Italy), according to Equation (3):

(3) $$\;R_{a}=\frac{1}{n}\sum_{i=1}^{n}\left|Y_{i}\right|$$

In this equation, *R_a_* represents the arithmetic mean of the absolute deviations of the evaluation profile (*Yi*) from the mean line.

The root mean square roughness (*Rq*) was calculated according to Equation (4):

(4) $$Rq=\sqrt{\frac{1}{n}}\sum_{i=1}^{n}Y_{i}^{2}$$

where *R_q_* is the root mean square of the profile deviations from the mean line, providing greater sensitivity to large surface irregularities than *R_a_*.

The average maximum height roughness (*Rz*) was calculated according to Equation (5):

(5) $$\;Rz=\frac{1}{5}\sum_{i=1}^{5}{(Y}_{pi}+\left\lfloor Y_{vi}\right\rfloor )$$

where *R_z_* represents the average maximum height of the profile, calculated as the mean of the sums of the heights of the five highest peaks (*p_i_*) and the depths of the five deepest valleys (*v_i_*) within the evaluation length.

The experimental values (listed in detail in Table S3 of the Supplementary Information) correspond to the average of three independent measurements obtained for each sample formulation.

#### 2.2.8. Shore-D Hardness

Surface Shore-D hardness was measured for PBSv–PBSr and PBSv–MIXr samples in accordance with the ASTM D2240 standard [29], using a Shore D durometer (model PCE TH210FJ, PCE Italia s.r.l., Capannori, Italy; resolution 0.1 Shore D units, accuracy ±1 within a scale range of 0–100) [30]. A 5 kg static mass was applied to the top of the durometer to ensure consistent indenter penetration. For each formulation, measurements were repeated ten times on the same specimen at different surface locations. The reported hardness values correspond to the average of these measurements.

#### 2.2.9. Color Measurement

The colorimetric properties of the samples were determined using a PCE-CSM 1 colorimeter (PCE Instruments, Manchester, UK). The measurements were performed in the CIELAB color space, where L* denotes lightness (0 = black, 100 = white), while a* and b* describe the green (−) to red (+) and blue (−) to yellow (+) chromatic axes, respectively [31]. For each sample, ten measurements were collected at different locations to ensure reproducibility and reduce the influence of local surface heterogeneity, and the reported values correspond to their average. The total color difference (ΔE*) was calculated from the obtained L*, a*, and b* values according to Equation (4):

(6) $$\;∆E*={\left[{\left(∆L*\right)}^{2}+{\left(∆a*\right)}^{2}+{\left(∆b*\right)}^{2}\right]}^{1/2}$$

where L*, a*, and b* represent the color coordinates of the reference sample. The values of lightness (L*) and of Δ*E*, listed in Table S3 in Supplementary Information, represent the average of these measurements. To reproduce the actual appearance of the samples, the CIELAB coordinates were converted into the CIEXYZ color space and subsequently into linear RGB values proportional to light intensity. A gamma correction was then applied to obtain the final display RGB values. Additional color data are provided in Table S3 of the Supplementary Information.

#### 2.2.10. Scanning Electron Microscopy (SEM)

SEM analysis: fractographic characterization of the tensile-tested dog-bone specimens was performed by SEM using a ZEISS Crossbeam 540 instrument (Carl Zeiss Microscopy GmbH, Jena, Germany). Images were collected at 1000× magnification with an operating voltage of 5 kV. Before examination, the fracture surfaces were metalized with a chromium coating using a Quorum Q150T ES sputtering system (Quorum Technologies, West Sussex, UK) to enhance the electrical conductivity of the specimens. The optical microscopy (OM) images were collected using a Hirox KH8700 digital microscope (Hirox, Tokyo, Japan) coupled with an MX(G)-5040Z lens (Hirox Co., Ltd., Tokyo, Japan).

## 3. Results

### 3.1. Assessment of Prototype Suitability for the Intended Application

**Figure 3:**
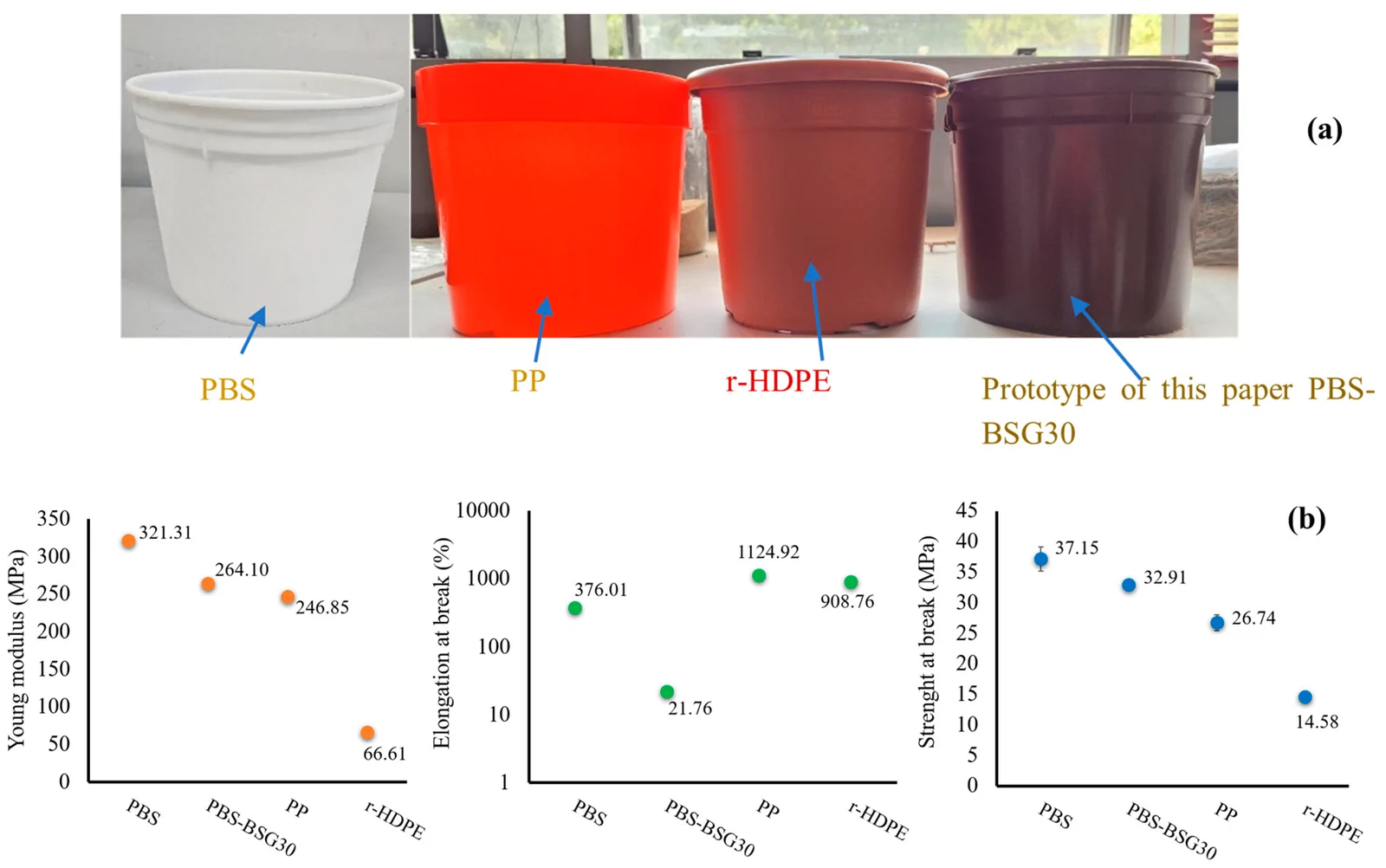
(**a**) Photos of commercial injection-molded plant pots made in polybutylene succinate (PBS), polypropylene (PP), recycled high-density polyethylene (r-HDPE), and a prototype of PBS-BSG30 investigated in this paper (**b**). Mechanical tensile comparison of the tensile parameters of the plant pots: Young’s modulus (E), tensile strength at yielding (σ_y_) and at break (σ_r_), elongation at break (ε_r_).

At the time of production (February 2024), the mechanical tensile properties of the injection-molded PBS-BSG30 bio composite pots were compared with those of commercially available fossil-based plastic pots (currently used in horticultural applications), made from recycled high-density polyethylene (r-HDPE) and polypropylene (PP), which were commercially purchased from DIRECT SICILY Mediterranean Plants (Via Petrarca, C.da Salicà, S. Biagio, 98050 Terme Vigliatore (ME)), as well as with neat polybutylene succinate (PBS). Dog-bone specimens were machined directly from the pots (shown in Figure 3a) and subjected to tensile testing; the results are reported in Figure 3b.

The analysis of the main mechanical parameters indicates that the PBS-BSG30 prototype exhibits satisfactory tensile performance. It shows a tensile strength at break of 37.15 MPa, exceeding the values measured for both PP and r-LDPE, and a stiffness of 264.10 MPa, which is also higher than that of commercial fossil-based materials. These properties, combined with the biodegradability of the PBS-BSG30 bio composite [20], provide a significant advantage by enabling a more sustainable product life cycle. The only limitation is its lower elongation at break (21.76%), which is more than one order of magnitude lower than that of PP and r-LDPE (1125% and 909%, respectively). However, such a high degree of deformability is not required for the intended application as a plant pot and therefore does not represent a practical drawback. For this specific application, it will also be necessary to validate the biological interaction of the bio-composite within the soil, investigating its potential effects on the biota and verifying any impact on plant germination and growth processes.

### 3.2. Processability: Torque Analysis

**Figure 4:**
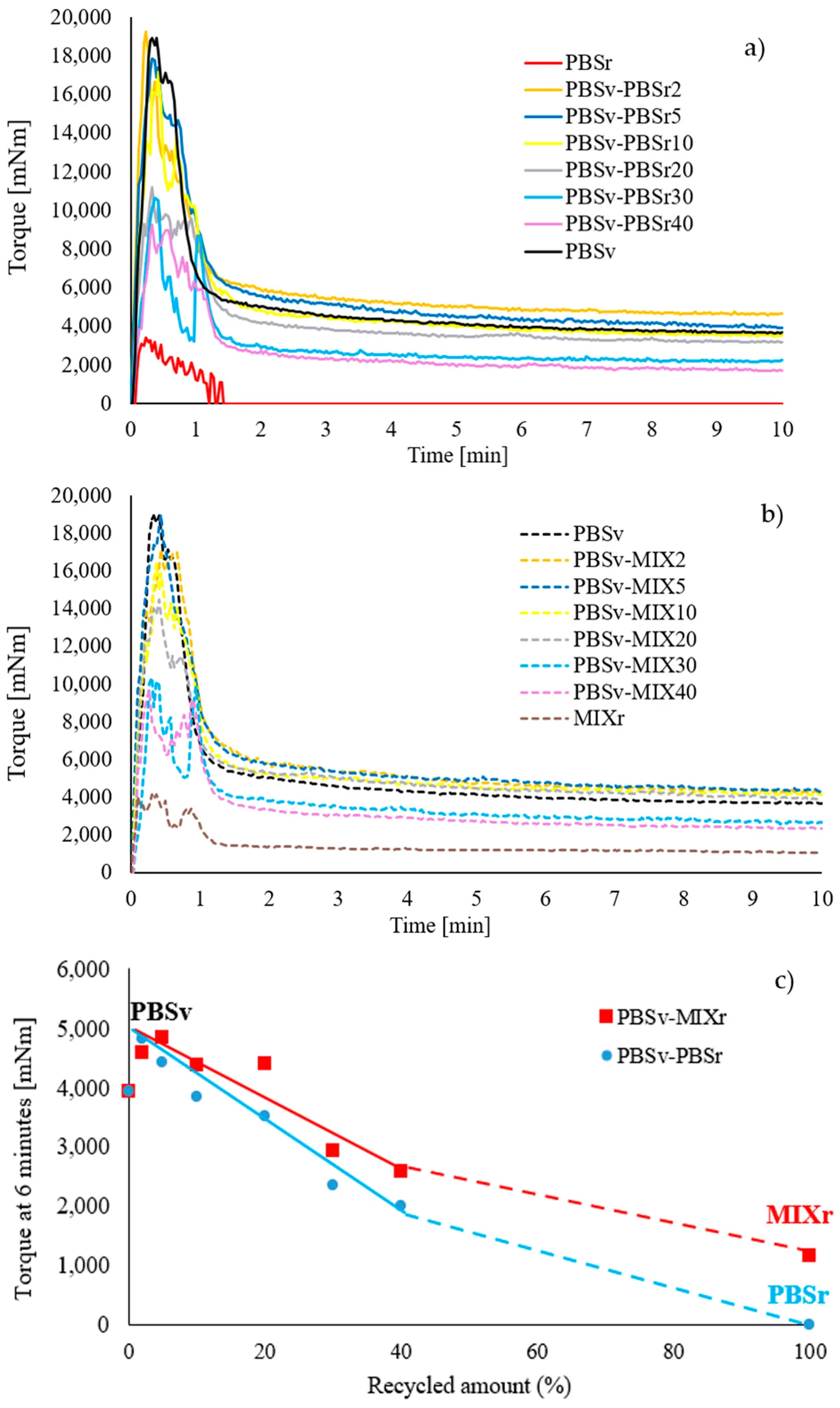
Torque curves vs. mixing time of all the studied samples: PBSv, PBSr, and PBSv-PBSr (**a**), and PBSv, MIXr, and PBSv-MIXr (**b**). Torque after 6 min of PBSs-PBSr and PBSs-MIXr vs. the percentage of recycled amount (**c**).

Torque–time curves over the ten-minute mixing period are presented in Figure 4a,b. An initial peak in torque is observed upon the addition of the materials to the mixing chamber. Subsequently, the torque for all mixtures decreases and stabilizes after approximately 5–6 min, indicating that the components have fully blended.

As the maximum torque peak consists of multiple sub-peaks due to the progressive melting of the material upon addition to the chamber, torque values after 6 min of mixing were selected for analysis. The torque values at 6 min are detailed in Table 2 and illustrated in Figure 4c, where the reference materials (PBSv, PBSr, and MIXr) are indicated with colored arrows in all graphs. The torque of virgin PBS (3940 mNm) increases with the addition of small amounts of recycled PBSr and MIXr materials (<10 wt.%).

The incorporation of recycled PBS into a virgin matrix generally reduces processing torque and melt viscosity [32]. Torque values for PBSv–MIXr blends are generally higher than those for PBSv–PBSr blends at the same recycled content, in line with previous studies reporting that the addition of agri-food waste from brewer’s grains increases the viscosity of the molten polymer [16] due to its lignin–cellulosic nature.

From a chemical point of view, PBS is a polyester, like polyethylene terephthalate (PET), with ester groups along the main chain. However, unlike PET, which contains rigid aromatic units, PBS has an aliphatic backbone that imparts greater flexibility and significantly lower glass-transition and melting temperatures. In the case of PET, the addition of recycled material to virgin matrices generally results in a reduction or maintenance of processing torque, attributable to polymer chain scission induced during recycling and the consequent decrease in melt viscosity. Under certain conditions, however, torque increases may be observed, related to molecular reconditioning processes or chain extension reactions that promote the formation of branched structures and an increase in molecular weight [33].

Like PET, when PBS is subjected to thermomechanical stress, it could generate radical species that evolve either towards chain scission (at high stress) or towards recombination reactions (at lower stress), leading to the formation of branched structures [11,34]. Consequently, the rheological response of melt torque could be governed by the competition between degradation via chain scission and branching phenomena arising from non-selective radical recombination [24,35]. In our specific system consisting of blends of virgin PBS with PBSr and MIXr (containing BSG), the variation in melt torque is governed by the balance between phenomena in the recycled PBS fraction and effects arising from the presence of BSG in MIXr.

However, the torque trend appears similar across the two blend types: at low recycled content of PBSr and of MIXr (<10 wt.%), the torque increases from 3940 mNm in PBSv to 4829 mNm in PBSv-PBSr2 and to 4853 mNm in PBSv-MIXr5, suggesting that chain branching predominates. At contents above 20 wt.%, a decrease in torque is observed in both blends (up to 1993 mNm in PBSv-PBSr40 and up to 2590 mNm in PBSv-MIXr40), indicating that chain scission becomes the dominant mechanism. The two linear trends checked up to 40 wt.% converge towards the limiting torque values of the neat materials, namely 1161 mNm for MIXr and 0.46 mNm for PBSr (see light-dotted lines in Figure 4a). These latter very low torque values of PBSr and MIXr compared to all other materials agree with the MFR data discussed previously (listed in Table 1) and confirm that these materials have undergone some degradation during the past two years of post-production warehouse storage (reasonably due to photodegradation and/or exposure to moisture) [36].

The interpretation of the degradation mechanism, in particular the distinction between chain scission and branching phenomena, remains hypothetical at present, in the absence of direct data obtained through further analytical techniques (e.g., GPC/SEC-gel permeation chromatography-GPC, also known as size exclusion chromatography -SEC, and/or rheological analyses).

### 3.3. Mechanical Performance: Tensile Test

**Figure 5:**
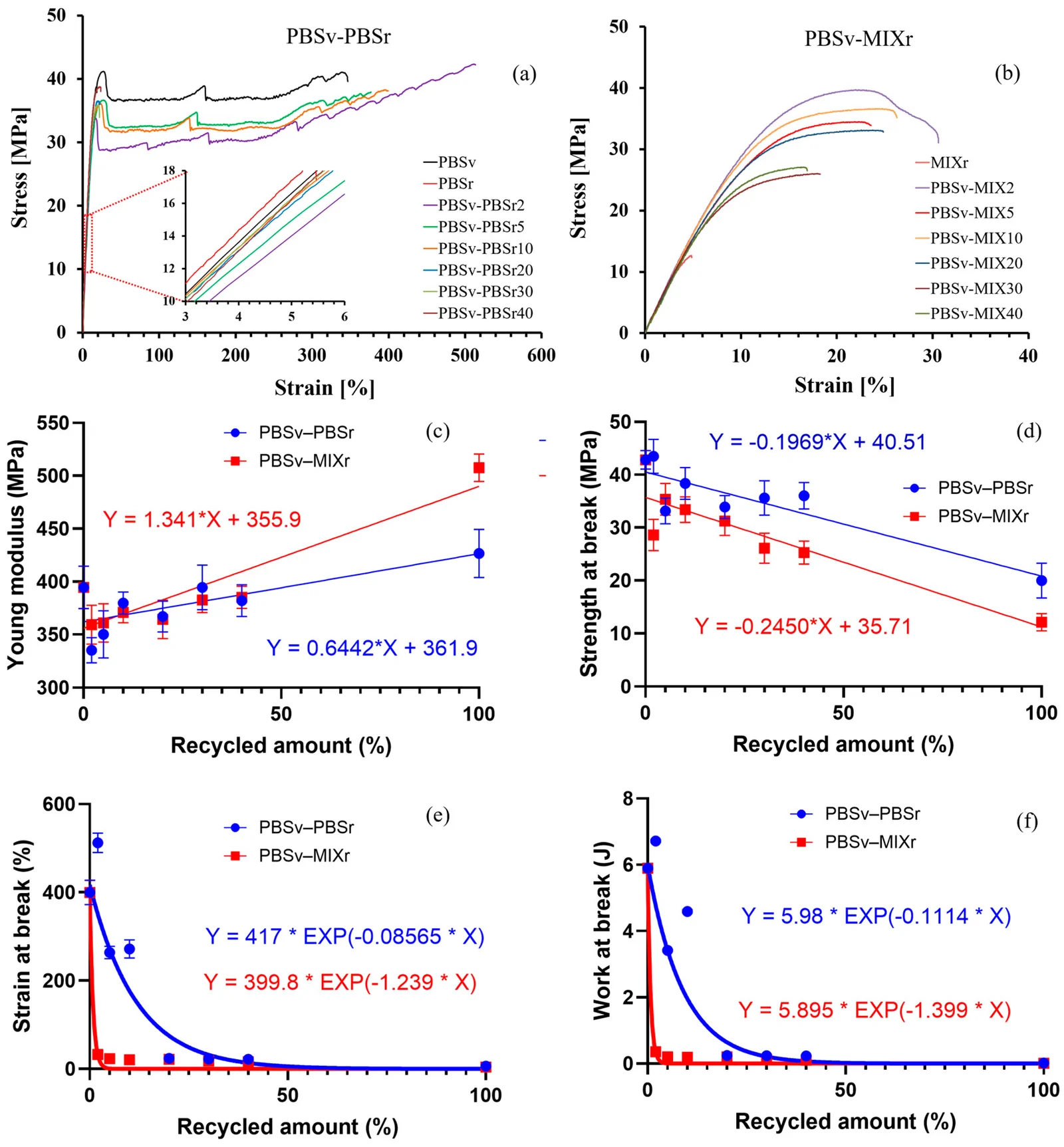
Stress–strain curves of PBSv-PBSr blends (**a**) and of PBSv-MIXr blends (**b**). Young’s modulus (**c**), elongation at break (**d**), strength at break (**e**), and work at break (**f**) of PBSv-PBSr blends and of PBSv-MIXr blends. The symbol “ * ” in the equations is the mathematical symbol for multiplication.

Table S1 (Supplementary Information) reports the detailed values of the mechanical parameters derived from the stress–strain curves of the PBSv/PBSr blends (Figure 5a) and PBSv/MIXr blends (Figure 5b). The stress–strain curves clearly show that adding PBSr to virgin PBS at 2–10 wt.% preserves the material’s ductile character, despite PBSr’s intrinsic brittleness, as suggested by the MFR and torque results discussed before. The yield strength of PBSv remains essentially unchanged (~39 MPa) with the inclusion of 10 wt.% PBSr (*p* > 0.9999), whereas the yield strain decreases from approximately 31% to ~24% (*p* < 0.0001). Other mechanical parameters, including stiffness, break tensile strength, break strain, and work at break, show progressive reductions: stiffness decreases from ~394 MPa to ~380 MPa (reduction of ~3.5%, *p* = 0.6009), break strength from ~43 MPa to ~38 MPa (reduction of ~11% *p* = 0.0004), break strain from ~399% to ~271% (reduction of ~32%, *p* < 0.0001), and work at break from ~5.9 J to ~4.6 J (reduction of ~22%, *p* < 0.0001). When higher amounts of recycled PBSr are added to virgin PBS (20–40 wt.%), no yielding occurs, and the ultimate strain decreases by approximately an order of magnitude, reaching around 24%. The tensile strength decreases from ~43 MPa for PBSv to ~36 MPa for PBSv–PBSr40.

The trends of Young’s modulus, tensile strength, elongation at break, and work at break as a function of recycled content are presented in Figure 5c–f. The data indicate that the material stiffens with increasing recycled content (Figure 5c), while tensile strength, elongation, and work at break progressively decrease (Figure 5e,f). Across all mechanical parameters, the PBSv–PBSr blends consistently show higher values than the PBSv–MIXr blends. This suggests that the addition of MIXr destabilizes the virgin polymer matrix more than recycled PBSr.

PBSv–MIXr blends exhibit low ductility even at minimal MIXr content (2 wt.%), with a stiffness of 360 MPa, tensile strength of 28.61 MPa, elongation at break of 33%, and work at failure of 0.35 J. In the blend with the highest MIXr content (PBSv–MIXr40), stiffness increases by ~7% to 385 MPa, tensile strength decreases by ~12% to 25 MPa, elongation at break decreases by ~51% to 16%, and work at failure decreases by ~34% to 0.23 J, compared with PBSv–MIXr2.

Brewer’s spent grain (BSG) particles can contribute to the oxidative stability of the composite due to the presence of lignin, which acts as a natural antioxidant. However, the BSG filler exhibits limited interfacial adhesion with the polymer matrix, since no specific compatibilizing agent was incorporated into the bio composite formulation.

### 3.4. Comprehensive Statistical Analysis of the Mechanical Properties: Two-Way ANOVA, Multiple Comparisons and Regression Modeling

This section includes the assessment of data normality and homogeneity of variances, two-way ANOVA, Bonferroni’s multiple comparisons, effect size analysis (partial η^2^), and regression modeling of the tensile mechanical properties.

To comprehensively evaluate the influence of recycled material and recycled content on the tensile behavior of the investigated blends, a statistical workflow combining assumption testing, inferential statistics, and regression modeling was performed. Detailed statistical outputs, including normality tests, homogeneity of variance tests, two-way ANOVA, Bonferroni’s multiple comparisons, effect size analysis (partial η^2^), and regression analyses, are reported in the Supplementary Information (See the “Comprehensive statistical analysis of mechanical tensile properties” in the Supplementary Information).

The normality of the experimental data was first verified using the Shapiro–Wilk test, while homogeneity of variances was assessed using the Brown–Forsythe test. Since some fracture-related properties exhibited heteroscedasticity, Welch’s ANOVA was additionally performed to verify the robustness of the statistical conclusions. The agreement between the conventional and Welch ANOVA confirmed that the observed differences among the formulations investigated were not affected by unequal variances (Tables S6 and S7 in Supplementary Information).

The effects of material type (PBSr versus MIXr), recycled content, and their interaction on the mechanical properties were subsequently evaluated by two-way ANOVA, whereas Bonferroni’s multiple comparisons test was used to identify statistically significant differences between individual formulations. In addition to statistical significance, partial eta squared (partial η^2^) was calculated to quantify the magnitude of each experimental factor, allowing the relative contribution of material type, recycled content, and their interaction to be assessed independently of the residual variance (Tables S8 and S9 in Supplementary Information).

Yield stress (σy) and yield strain (εy) were determined only for the PBSv–PBSr formulations containing up to 10 wt.% recycled PBS, whose stress–strain curves exhibited a well-defined yielding point followed by plastic deformation and strain hardening (Figure 5a). In contrast, the PBSv–MIXr blends, as well as the PBSv–PBSr formulations containing 20–40 wt.% recycled PBS, did not exhibit a distinct yield point (Figure 5a,b). Instead, these materials fractured before the onset of stable plastic flow, displaying a predominantly brittle tensile response. Consequently, σy and εy could not be experimentally determined for these formulations. Because yield data were not available across all combinations of the experimental factors (material type × recycled content), a balanced factorial two-way ANOVA could not be performed for σy and εy. Therefore, the statistical evaluation of these two parameters was limited to the preliminary assessment of the assumptions required for parametric analyses, namely the Shapiro–Wilk normality test and the Brown–Forsythe homogeneity of variances test, the results of which are reported in Tables S2 and S3 (Supplementary Information). No factorial ANOVA, post hoc multiple comparisons or regression analyses were carried out for σy and εy, since these parameters are not physically meaningful for formulations that do not exhibit yielding during tensile deformation.

Since recycled content represents a continuous quantitative variable, complementary regression analyses were performed to describe the evolution of the mechanical properties as a function of recycled fraction. Linear regression models were applied to Young’s modulus and stress at break, whereas exponential decay models provided the best fit for elongation at break and work at break. Comparisons of regression slopes and intercepts were also performed to evaluate differences between the PBSv–PBSr and PBSv–MIXr systems (Table S10 in Supplementary Information).

Overall, the combined statistical approach demonstrated that recycled content is the primary factor controlling the evolution of elastic stiffness, whereas fracture-related properties are strongly influenced by both recycled content and material type, as well as by their interaction. The regression analysis further demonstrated that stiffness changes approximately linearly with increasing recycled content, whereas ductility and toughness undergo a pronounced non-linear deterioration, providing complementary evidence for the progressive embrittlement induced by thermo-mechanical recycling.

### 3.5. Morphological Observations

**Figure 6:**
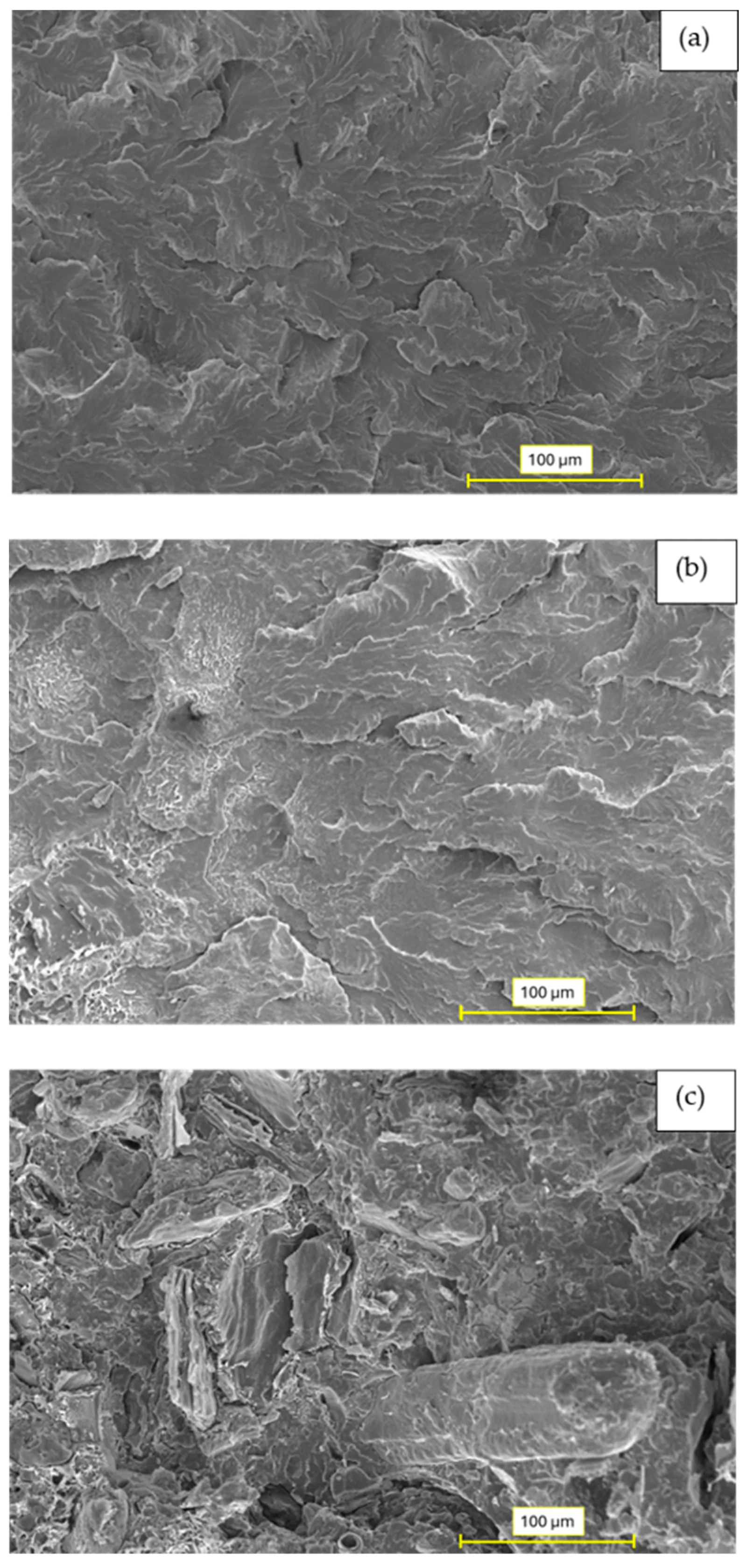
SEM microscopy at 1000× of PBSv (**a**), PBSr (**b**), and MIXr (**c**).

Mechanical reprocessing involved in recycling promotes the formation of microcracks and voids, as clearly evidenced by SEM morphological analysis (Figure 6). These defects act as preferential pathways for the penetration of moisture and other environmental agents, thereby accelerating material deterioration and contributing to the observed decline in both mechanical and physical performance. In particular, the highly hydrophilic nature of BSG promotes moisture absorption, further promoting the degradation process of the biocomposite.

Figure 6a shows the surfaces of PBSv, PBSr, and MIXr. Both PBS surfaces (virgin and recycled) are rough, as is typical for a ductile and deformable continuous-phase material. However, the morphology of MIXr is different due to the presence of elongated BSG rods protruding from the matrix and the presence of voids between the reinforcement and the matrix, due to the absence of a compatibilizing agent in the mixture.

**Figure 7:**
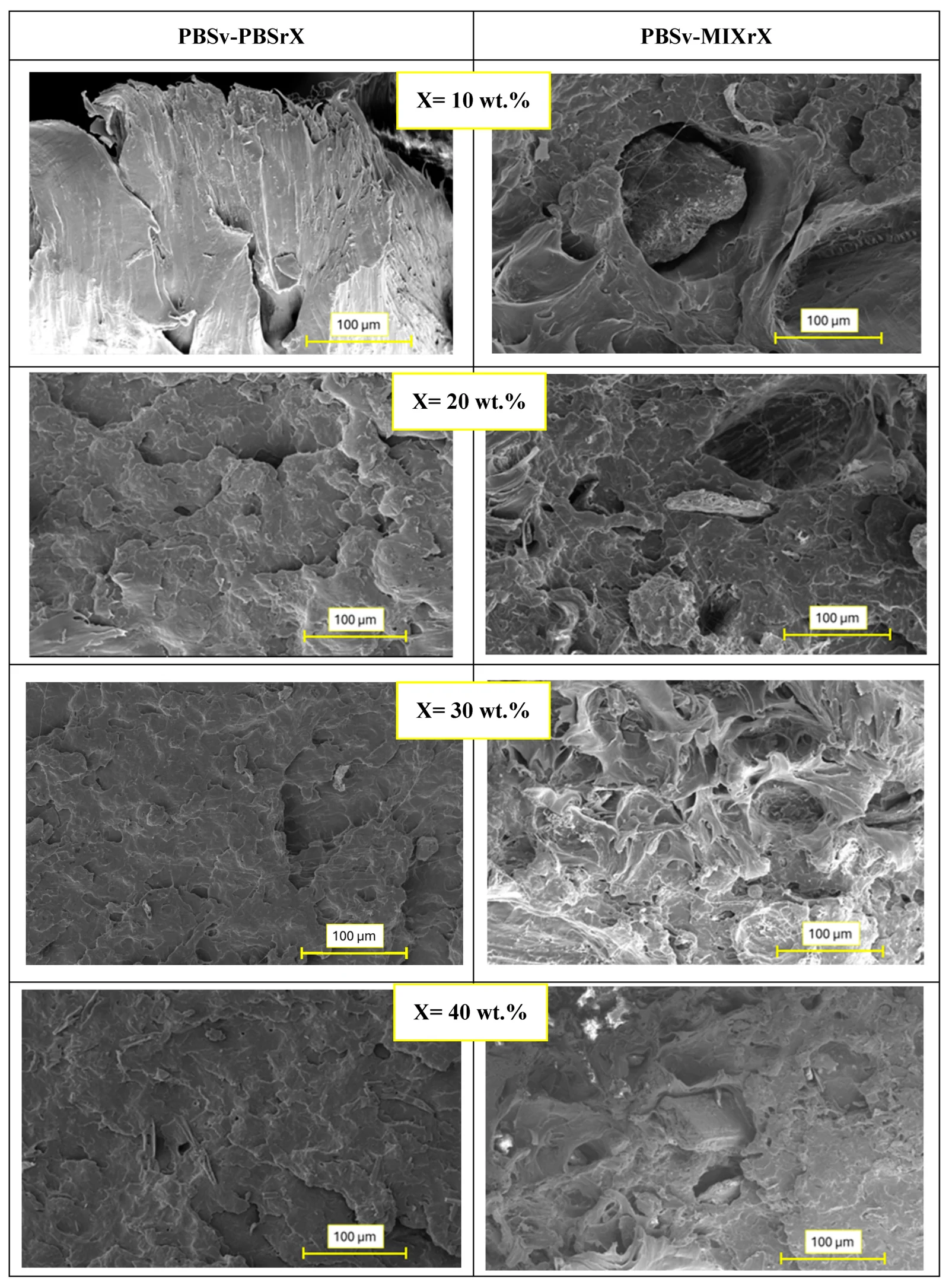
SEM Analysis at 1000× of PBSv-PBSr X (**left column**) and PBSv-MIXr X (**right column**).

Figure 7 shows the surfaces of the PBSv-PBSr and PBSv-MIXr blends at different percentages of recycled material (from 2 wt.% to 40 wt.%). The morphology of virgin PBS/recycled PBS blends is always characterized by a rough fracture surface, regardless of the percentage of recycled material. Only in the blend containing 40% recycled PBS by weight are some limited void areas observed.

In contrast, PBSv-MIXr blends consistently show the presence of BSG particles and an overall smoother fracture surface than PBSv-PBSr blends. Localized voids at the interface between the BSG particles and the polymer matrix are clearly visible, indicating poor interfacial adhesion. Furthermore, numerous indentations are observed left by the detachment of the BSG particles during the brittle fracture induced by the mechanical tensile test. The presence of voids, cracks, and particle pull-out phenomena confirms the brittle behavior of these blends.

A filler can effectively reinforce the polymer only when it is well bonded to the matrix, allowing efficient stress transfer throughout the material. In contrast, the interfacial voids clearly observed in the SEM micrographs hinder stress transfer, causing the filler to behave as an inclusion or defect rather than as a reinforcing phase. To overcome these limitations, ongoing research involves the development of composites compatibilized with natural compatibilizing agents. This approach is expected to improve interfacial adhesion between the filler and the matrix, reduce interfacial tensions, and ultimately improve the durability and recyclability of the material. The only formulation developed by our research group that included a compatibilizing additive employed α-tocopherol (vitamin E), whose action is primarily based on physical interactions and is therefore relatively weak [23]. Future studies should focus on the development and evaluation of other bio-based and environmentally friendly compatibilizers capable of providing a more effective compatibilizing action.

Based on these results, we can conclude that virgin polybutylene succinate can be blended as follows:

(a) with PBSr in an amount up to 10 wt.%, to maintain good ductility, with a yield stress of ~35–39 MPa, yield strain around 25%, and elongation at break near 270%. Such blends are suitable for second-life materials used in applications requiring good deformability and toughness. Higher recycled content in PBSr eliminates the ductility of virgin PBS, resulting in an impressive reduction in strain to 22–24%.

(b) With MIXr in an amount up to 30–40 wt.%, producing blends that are brittle but suitable for second-life materials that can find applications demanding high stiffness, low deformability, and low toughness. MIXr is intrinsically stiff and brittle, with an elongation at break of ~4%. Adding MIXr to PBSv reduces deformability by roughly 17–25 times, with elongation at break decreasing from nearly 400% to 23–16% as MIXr content increases from 2 wt.% to 40 wt.%, respectively. This is due to the presence of BSG, which disturbs the structural order within the polymer matrix and acts as a filler rather than a reinforcing agent [16]. This results in a higher stress concentration at the filler–matrix interface, where voids and discontinuities form [12].

### 3.6. Thermal Stability

**Figure 8:**
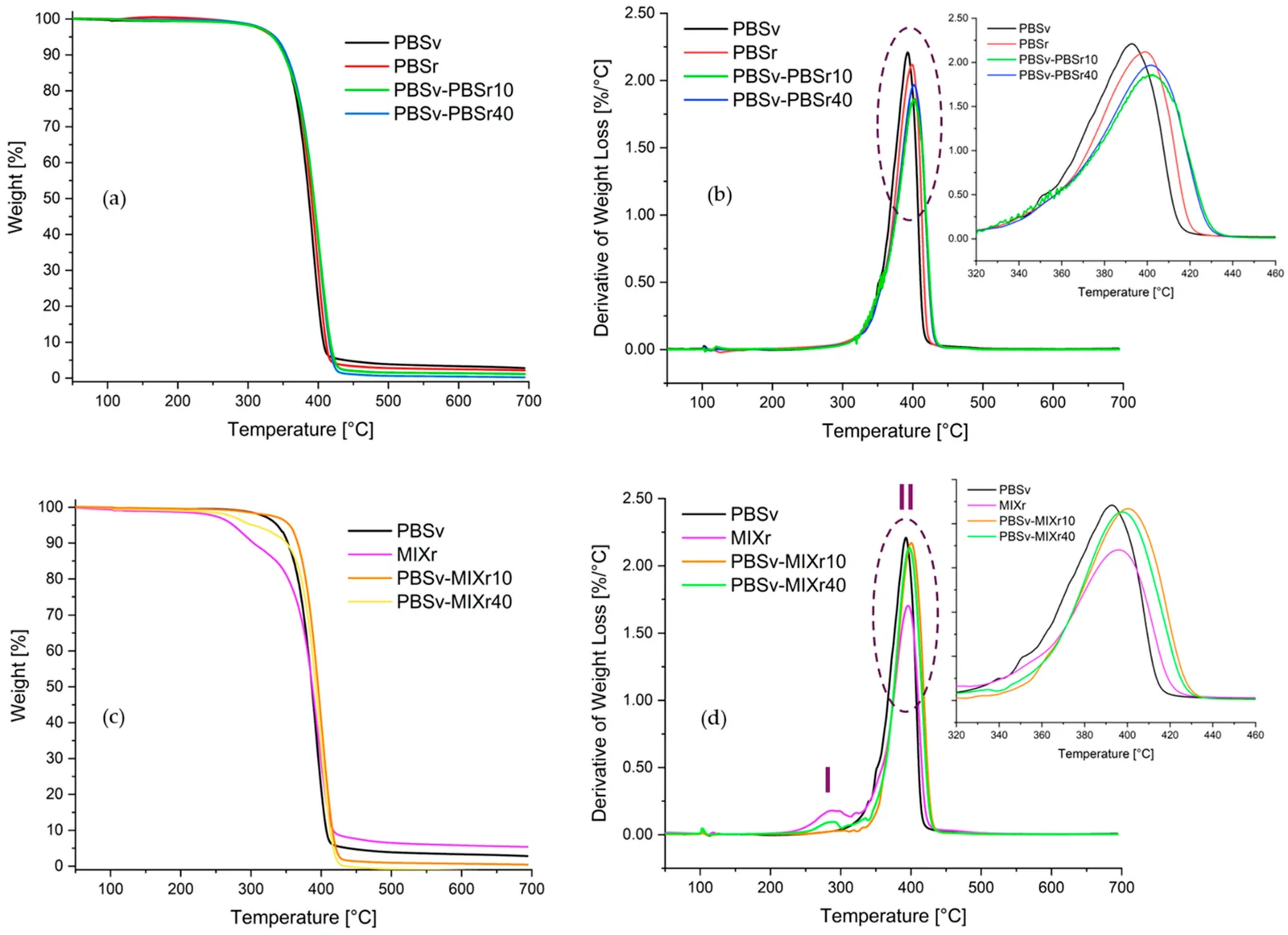
TGA (**a**,**c**) and DTGA (**b**,**d**) of PBSv, PBSr, MIXr, and of PBSv/PBSr and PBSs-MIXr blends.

To evaluate the thermal stability of the blends, thermogravimetric analysis (TGA) was performed. The TGA and DTG curves are shown in Figure 8a,b, and the main quantitative parameters are summarized in Table S2 (in Supplementary Information). For clarity of curve visualization, only the PBSv-PBSr and PBSv-MIXr blends containing either a low (10 wt.%) or a high (40 wt.%) recycled fraction are discussed in detail.

The TGA curves show that the PBSv/MIXr blends exhibit different thermal stability compared to the PBSv/PBSr systems. In particular, the onset temperature (T_onset_) of virgin PBS is approximately 326 °C. The addition of recycled PBS results in a slight decrease in this parameter, which stands at around 318 °C for the PBSv-PBSr40 blend, a decrease of only 2.5%. Conversely, the introduction of MIXr results in a much more significant reduction in thermal stability: for the PBSv-MIXr40 blend, the T_onset_ of PBSv decreases from 326 °C to approximately 255 °C, a decrease of about 22%. Accordingly, the thermal stability order of the PBSv/MIXr blends can be summarized as follows:

PBSv > PBSv–MIXr10 > PBSv–MIXr40 > MIXr.

MIXr exhibits the lowest thermal stability, with a T_onset_ of approximately 238.95 °C. The DTGA curves show two main degradation peaks in the MIXr-containing samples. The first peak (Peak I), highlighted in Figure 8d, is attributed to the decomposition of brewer’s spent grains (BSG), while the second peak (Peak II) corresponds to the thermal degradation of PBS.

As expected, peak I is absent in PBSv–PBSr10 and PBSv–PBSr40 because these formulations do not contain BSG. In the PBSv-MIXr10 and PBSv-MIXr40 samples, the first peak appears in the 296–307 °C range, whereas the second peak, associated with PBS degradation, occurs between approximately 398 and 401 °C, in line with the behavior of neat PBSv (Table 3).

These results indicate that the lower T_onset_ values observed for the PBSv–MIXr blends are mainly due to early degradation of the BSG-containing phase, while the principal PBS degradation event remains close to that of virgin PBS. Overall, these results confirm that PBSv-MIXr blends are thermally less stable than PBSv-PBSr blends.

To quantitatively assess the effect of BSG on the thermal stability of the biocomposite, kinetic parameters should be determined using established thermal degradation models, such as the Kissinger, Ozawa–Flynn–Wall, or Friedman methods [37,38]. Such analyses are beyond the scope of the present work and will be addressed in future studies.

### 3.7. FTIR Analysis

The FTIR transmittance spectra of PBSv, PBSr, MIXr, BSG, and blends of PBS with low (10 wt.%) and high (40 wt.%) BSG amounts (PBSv-PBSr10–40 and PBSv-MIXr10–40) were collected in the 4000–500 cm^−1^ range (Table S2 Supporting Information). Peak assignments are reported in Table 3, with the main absorption bands identified by alphabetical labels. We considered the stretching (symmetric and asymmetric) and the bending of hydroperoxide, hydroxyl, and carbonyl signals. As is known, PBS is an aliphatic polyester containing carboxylic groups in its chemical structure that is obtained by the polymerization of succinic acid and 1,4-butanediol. BSG is mainly composed of cellulose, hemicellulose, lignin, and proteins [39]. Hence, the chemical structure is made up of several glucose molecules characterized by the content of numerous hydroxyl groups. The overlapping of the main peaks of the FTIR spectrum occurs, in general, in all the samples investigated. In detail, we compared the FTIR spectra of virgin and recycled PBS, of MIXr, PBSv, and BSG (Figure S1), and of PBSv, PBSr, PBSv-PBSr10/40, and PBSv-MIXr10/40. These results indicate that the recycling process does not significantly affect the chemical qualitative structure of PBS, as no new functional groups are observed beyond those present in the virgin material.

The unchanged chemical composition revealed by FTIR analysis suggests that the differences observed in physical properties might be mainly attributable to the structural arrangements of the blends, rather than to chemical modifications induced by the recycling process. These findings are consistent with the literature, which reports that PBS retains its chemical structure during processing and recycling, with no formation of new functional groups [35]. The same authors emphasize that variations in mechanical properties are primarily driven by changes in chain mobility, molecular weight, and the physical structure of the material, rather than by chemical transformations [40]. In this context, it could be of interest to investigate potential variations in molecular weight and its distribution induced by degradation during recycling. These aspects, not addressed in the present work, will be the subject of future investigations.

**Figure 9:**
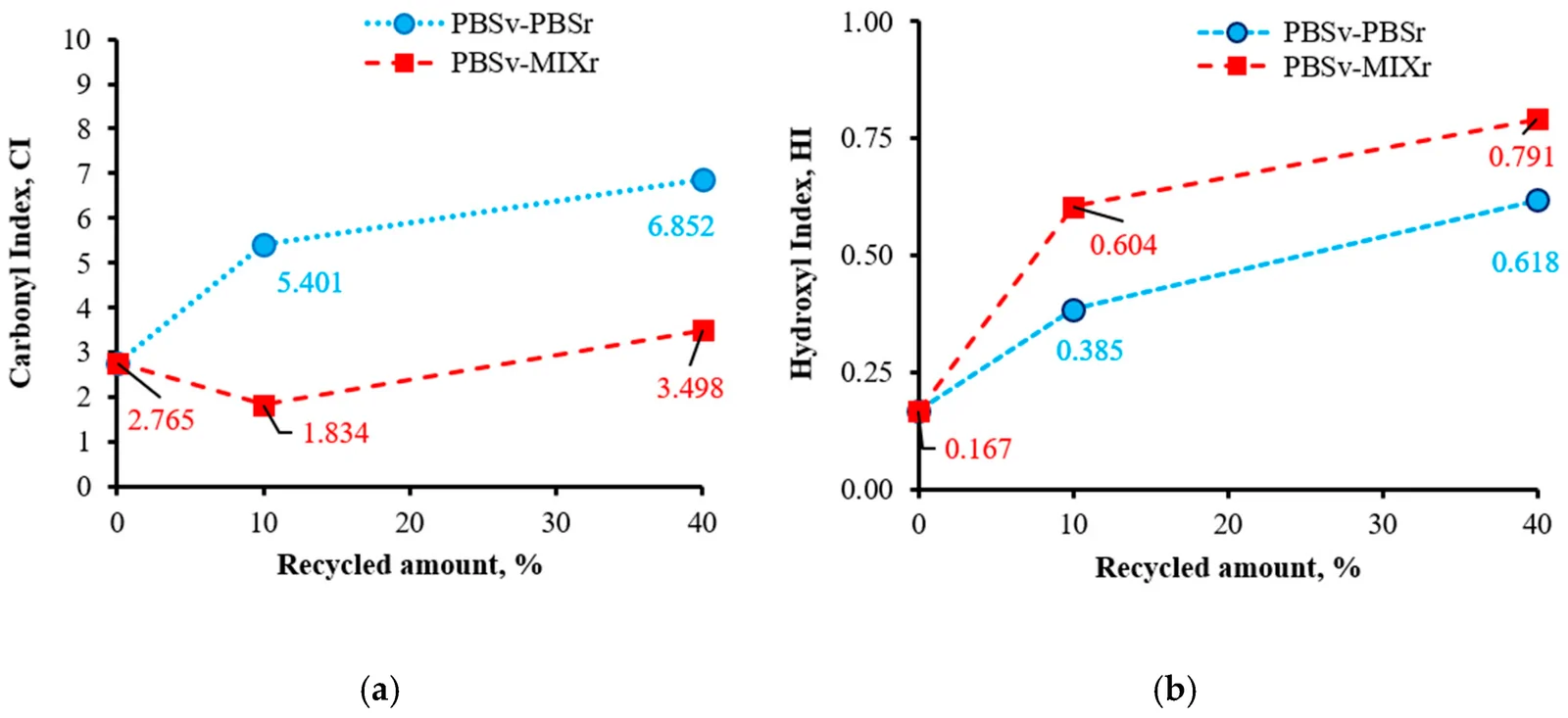
Carboxyl index (**a**) and hydroxyl index (**b**) vs. recycled amount.

FTIR analysis can highlight the effect of recycling on PBS by evaluating the change in the amount of the oxidized species as a function of the recycled amount. With this aim, we estimated the quantitative changes occurring in the chemical species amount during the recycling process by considering the Carbonyl Index (CI) and Hydroxyl Index (HI) of the investigated blends, according to Equations (1) and (2) reported in Section 2.2 (plotted in Figure 9). The trend of the graph shows an increase in carboxylic groups in both blends (PBSv–PBSr and PBSv–MIXr) as the recycled content rises. However, this increase is less pronounced in the PBSv–MIXr system compared to PBSv–PBSr. This behavior can be attributed to the presence of agro-food waste, namely BSG, in the MIXr. BSG exhibits a heterogeneous lignocellulosic structure consisting of cellulose, hemicellulose, and lignin, mainly originating from the barley husk fraction [41]. The lignin is a phenolic macromolecule known for its antioxidant activity. Indeed, lignin extracted from brewer’s spent grain has been reported to exhibit radical scavenging capacity, contributing to the overall oxidative stability of the material [42]. In our case, the presence of BSG helps limit the formation of carboxylic species in the recycled material, according to the findings of other authors [16,38].

The situation is instead reversed with respect to hydroxyl groups. In this case, a higher formation is observed in the PBSv–MIXr blends compared to PBSv–PBSr. This behavior can be attributed again to the presence of brewer’s spent grain (BSG). The high content of polysaccharides bearing hydroxyl groups, together with the porous morphology of the fibrous matrix, results in a significant presence of polar functionalities and, consequently, an overall affinity for water [43]. Therefore, the presence of BSG appears to promote a greater formation of hydroxyl species compared to PBSv–PBSr blends, where BSG is absent, in agreement with the literature data [12].

The variation in the CI/HI ratios could be associated with polymer chain scission phenomena; however, this interpretation remains indirect and should be verified through further analyses of the average molecular weight and molecular weight distribution. Future studies could therefore employ GPC-SEC to provide quantitative confirmation of any degradation resulting from chain scission.

### 3.8. Surface Features

Finally, an investigation of the surface features of all materials and blends has been performed by means of colorimetric, roughness, Shore D hardness, and SEM morphological analyses.

**Figure 10:**
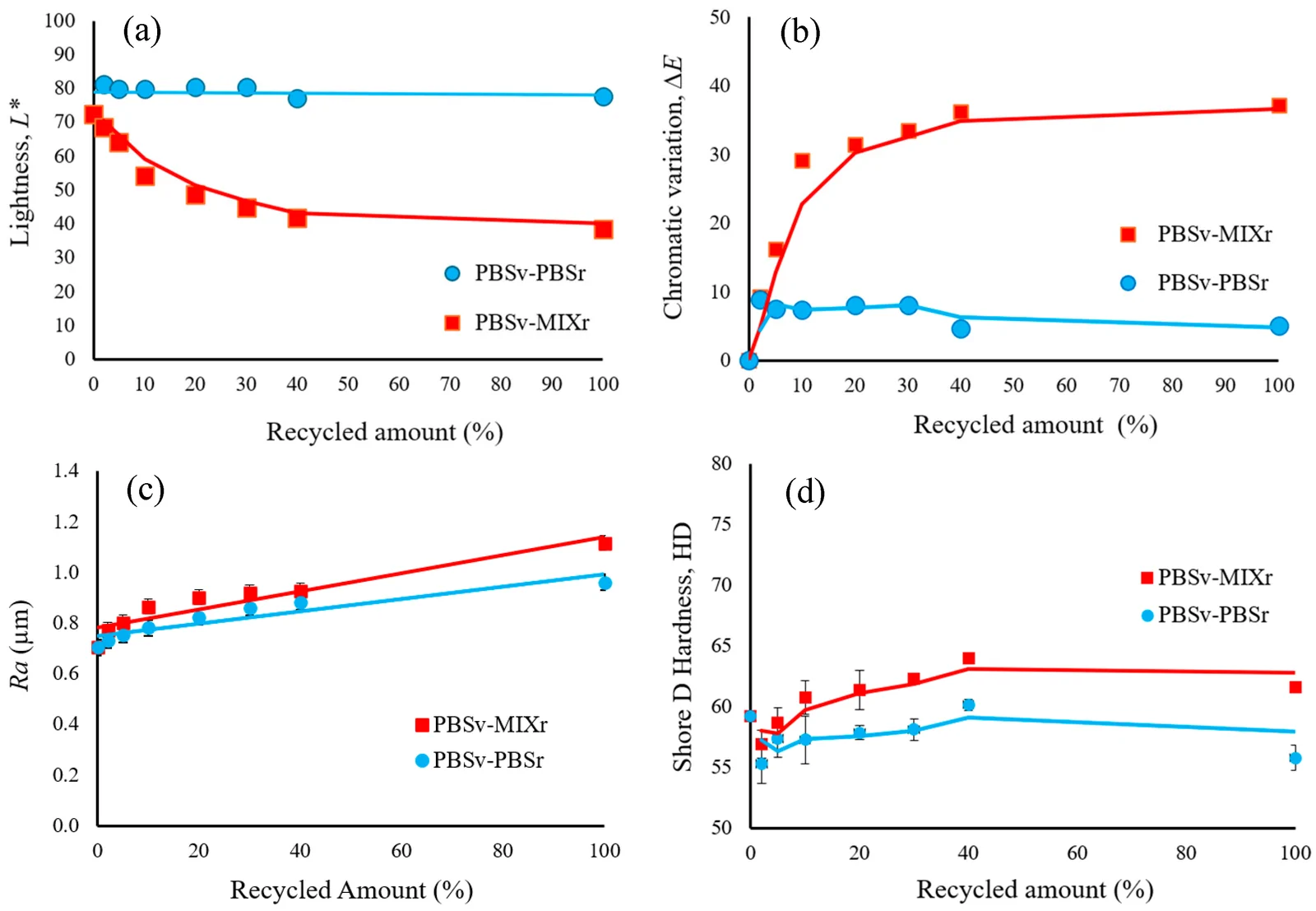
Lightness, L* (**a**), chromatic variation, ∆*E* (**b**), roughness, *Ra* (**c**), and Shore-D hardness (**d**), vs. the recycled amount, of all the sheets.

Lightness (L*) and chromatic variation (∆*E* value) as a function of the recycled amount are plotted in Figure 10a,b, respectively. The evaluation has been performed on the square-shaped sheets of the different blends plotted in Figure 2a,b. The evaluation procedure for color measurements is described in detail in the paper of Hejna et. al. [31].

The L* and ΔE* values of the white PBSv reference sheet were 72.55 and 0, respectively. The PBSv/PBSr blends exhibit only minor changes in lightness (from 72.55 to approximately 80) and color difference (ΔE*, from 0 to ~8) relative to the reference sheet of Figure 2a,b. In contrast, the PBSv/MIXr blends display a more pronounced variation in both parameters. The photographs reveal progressive darkening, from light brown tones to increasingly darker brown shades, as the proportion of MIXr in the blend increases, approaching the darkness observed in neat MIXr. Lightness (L*) decreases from 72.55 to 41.85 in PBSv/MIXr40 compared to PBSv (Figure 2a).

As previously discussed, the chromatic variation (ΔE*) of the PBSv/PBSr blends changes only slightly with the addition of recycled material. Conversely, the ΔE* of PBSv/MIXr blends increase substantially, reaching approximately 30 points relative to the initial color (Figure 2b). Similarly to the color results, surface roughness also changes in the blends compared to PBSv (Figure 2a). Detailed data are reported in Table S4 of the Supplementary Information. *Rz* and *Rq* data are reported in Table S5 of the Supporting Information for completeness. For the PBSv/PBSr blends, *Ra* surface roughness (Figure 10c) increased by ~4% in PBSv-PBSr2 (0.73 μm) and ~25% in PBSv-PBSr40 (0.88 μm), relative to PBSv (0.70 μm); see Figure 10c and Table S4. Greater variations were observed in the PBSv/MIXr blends: roughness increased by ~9% in PBSv-MIXr2 (0.77 μm) and ~32% in PBSv-MIXr40 (0.93 μm), relative to PBSv (0.70 μm). These differences are attributed to the presence of agro-food waste in MIXr, ranging from 0.6 wt.% to 12 wt.%, and to the higher degradation of the PBSv-MIXr compared to PBSv-PBSr blends. Hardness (Figure 10d) grows with the recycled amount, according to Young’s modulus discussed earlier, from 55.29 HD in PBSv-PBSr2 to 60.15 HD in PBSv-PBSr40 (+8.8%) and from 56.92 HD in PBSv-MIXr2 to 63.98 HD in PBSv-MIXr40 (+12.4%).

## 4. Conclusions

Fully biobased PBS pots offer several attractive features for horticultural applications and can serve as a sustainable alternative to conventional fossil-based polyolefin pots. Their recyclability has been investigated by reintroducing recycled material into a virgin PBS matrix after approximately two years of warehouse storage.

Two recycled fractions were considered: PBSr, obtained from neat PBS injection-molded pots, and MIXr, derived from PBS/BSG biocomposite pots containing 30 wt.% brewer’s spent grain (BSG). Both fractions were incorporated into virgin PBS (PBSv) at concentrations ranging from 2 to 40 wt.%, corresponding to a final BSG content of 0.6–12 wt.% in PBSv–MIXr blends. The resulting materials were evaluated in terms of processability, mechanical behavior, thermal and chemical stability, and surface properties.

The results demonstrate that BSG is the main factor limiting the recyclability of the material, as evidenced by both the variation in T_onset_ and the mechanical properties obtained from tensile tests.

PBSv–MIXr blends exhibit higher torque values than PBSv–PBSr systems because the lignocellulosic filler increases melt viscosity. At low recycled contents (2–5 wt.%), chain branching appears to prevail, leading to a torque increase, whereas higher amounts (10–40 wt.%) promote chain scission and a progressive torque reduction, indicative of degradation.

Incorporation of PBSr up to 10 wt.% largely preserves the ductile behavior of virgin PBS, while higher contents (20–40 wt.%) reduce ductility with minimal effect on thermal stability. In contrast, PBSv–MIXr blends exhibit rigid but little deformability (within 20–30% in the PBSv-MIXr2–20 formulations). Thermal stability progressively decreases: −16% in PBS-MIX10, and −22% in PBSv–MIXr40. This experimental evidence suggests that BSG disrupts the structural continuity of the polymer matrix, thus compromising the overall physical and mechanical performance of PBSv.

Comprehensive statistical analysis of mechanical tensile properties highlights that the effect of recycling depends on the mechanical property considered: Young’s modulus is primarily influenced by the recycled content, while fracture properties (σr, εr, and W) significantly depend on the type and amount of recycled material, as well as their interaction. Increasing the recycled fraction leads to a progressive reduction in molecular integrity and an increase in structural heterogeneity, resulting in worsening fracture toughness, ductility, and energy absorption. Recycled PBS better retains its original characteristics, while recycled MIX shows greater degradation and a more heterogeneous microstructure.

The conditions studied in this work represent a worst-case scenario, as the recycled raw material consisted of vessels that had undergone prolonged storage before reprocessing. Industrial recycling typically consists of production waste, such as burrs, edge trimmings, or defective parts, which are generally reprocessed immediately after production and are therefore not exposed to prolonged storage or physical aging. Consequently, these materials are expected to maintain superior quality and could potentially be incorporated into the virgin matrix at even higher percentages than those identified in this study. From this perspective, the recycling levels reported here can be considered conservative estimates, while more favorable recycling scenarios are likely achievable under typical industrial process conditions.

Recycling does not change the nature of the functional groups of PBS but modifies their concentration because carbonyl and hydroxyl groups increase with recycling. However, the antioxidant activity of lignin partially suppresses carbonyl formation, whereas the hydrophilic character of BSG cannot prevent the accumulation of hydroxyl species. Degradation also increases surface roughness and hardness, in agreement with the stiffness observed in tensile tests.

Although the incorporation of BSG is an effective strategy for the valorization of agro-industrial by-products and the development of sustainable biocomposites, it also introduces significant recycling challenges. Future research will focus on improving the interfacial compatibility between PBS and BSG through naturally derived compatibilizers, replacing conventional maleic-anhydride-based additives while preserving the fully biobased nature of the material and enhancing its long-term recyclability.

These results are particularly relevant for industrial applications in which commercial pots are recovered after prolonged use or storage. Considering that the European horticultural pot market accounts for several billion units annually, effective recycling strategies for these products could substantially reduce plastic waste and virgin polymer consumption, contributing to lower greenhouse gas emissions and supporting circular economy practices in the nursery sector. For a truly comprehensive assessment consistent with the principles of the circular economy, it would also be appropriate to consider the following aspects: material collection, sorting, and sorting infrastructure, potential contamination from soil, fertilizers, and other external agents, life cycle assessment (LCA), and economic feasibility analysis comparing mechanical recycling with industrial composting. These aspects, while fundamental to an overall assessment of the system’s sustainability, are beyond the scope of this study and therefore merit further investigation in future research.

## Figures and Tables

**Table 1:** Code, description, production and testing date, MFR, and melt density of the materials used in this research.

Code	Description	Production Date	Testing Date	MFR (g/10 min)	Melt Density (g/cm^3^)
PBSv	Virgin PBS	26 May 2022	24/11/2025	37.36 ±1.63	1.00 ± 0.02
PBSr	Recycled PBS	27 February 2024	25/11/2025	159.06 ±7.14	0.99 ± 0.13
MIXr	Recycled PBS (70 wt.%) + BSG (30 wt.%)	27 February 2024	17/11/2025	153.24 ± 29.71	1.20 ± 0.06

**Table 2:** Samples’ composition and torque.

Code	PBSv (%)	PBSr (%)	MIXr (%)	Amount of BSG in MIXr (%)	Amount of PBS in MIXr (%)	Torque After 6 min (mNm)
PBSv	100	-	-	-	-	3940 ± 12
PBSr	-	100	-	-	100	0.46 ±0.15
MIX	-	-	100	30	70	1161 ± 8
PBSv-PBSr2	98	2	-	-	-	4829 ± 8
PBSv-PBSr5	95	5	-	-	-	4415 ± 9
PBSv-PBSr10	90	10	-	-	-	3838 ± 12
PBSv-PBSr20	80	20	-	-	-	3524 ± 18
PBSv-PBSr30	70	30	-	-	-	2339 ± 8
PBSv-PBSr40	60	40	-	-	-	1993 ± 15
PBSv-MIXr2	98	-	2	0.6	1.4	4596 ± 11
PBSv-MIXr5	95	-	5	1.5	3.5	4853 ± 17
PBSv-MIXr10	90	-	10	3	7	4376 ± 10
PBSv-MIXr20	80	-	20	6	14	4401 ± 16
PBSv-MIXr30	70	-	30	9	21	2927 ± 6
PBSv-MIXr40	60	-	40	12	28	2590 ± 18

v = virgin, r = recycled, MIX = PBS (70 wt.%) + BSG (30 wt.%).

**Table 3:** FTIR signal peaks of PBS and BSG.

BSG			PBS		
Signal	Wavenumber (cm^−1^)	Attribution	Signal	Wavenumber (cm^−1^)	Attribution
**Interval**	3600–3200	Hydroperoxide, hydroxyl groups	B	2962	v_s_ (CH)
A	3281	v_s_ (OH)	D	2857	v_s_ (CH)
C	2920	v_s_ (CH)	F	1713	v_as_ (C=O)
E	2852	v_s_ (CH)	I	1473	δ (CH) in (CH_2_)
G	1736	v_as_ (C=O)	L	1152	v_as_ (C-O)
H	1630	v_s_ (C=O)	M	1044	v_s_ (C-O)
N	1032	δ (C-O)			

v_s_= symmetric stretching; v_as_ = asymmetric stretching; δ = bending.

## Data Availability

The data presented in this study are available on request from the corresponding authors.

## Supplementary Materials

The following supporting information can be downloaded at: https://www.mdpi.com/article/10.3390/polym18172119/s1, Figure S1. Comparison of FTIR Spectra of PBSv and PBSr (a), of PBSv, BSG, and MIXr (b), of PBSv, PBSr, MIXr, PBSv-MIXr10, PBSv-MIXr40, PBSv-PBSr10, and PBSv-PBSr40; Table S1. Mechanical parameters of all materials, pure and blended, processed by melt mixing: Young modulus (E), tensile strength at yielding (σy) and at break (σr), elongation at yielding (εy) and at break (εr), work at break (W); Table S2. TGA parameters of PBSv, PBSr, MIXr, and selected blends containing 10 wt.% and 40 wt.% of PBSr or MIXr; Table S3. Color parameters of PBSv-PBSr and of PBSv-MIXr blends; Table S4. Surface roughness (Ra) and percentage increase in roughness compared to virgin PBS (∆) of PBSv, PBSr, MIXr and all the blends; Table S5. Surface roughness parameters *Rq* and *Rz* of the PBSv/MIXr and PBSv/PBSr blends; Table S6. Summary of the test statistic of the Shapiro–Wilk normality test; p, probability value, performed for the investigated tensile properties; Table S7. Statistical summary normality test Brown-Forsythe ANOVA test or Welch’s ANOVA; p, probability value; Table S8. Complete two-way ANOVA results (Bonferroni’s multiple comparisons test); Table S9. Mean difference ANOVA results (Bonferroni’s multiple comparisons test); Table S10. Regression analysis of the mechanical properties.
