# Supplementary material for: Recycling Poly(Butylene Succinate)/Brewer’s Spent Grain Pot Prototypes
Source: Polymers (Basel). 2026 Aug 31;18(17):2119. doi: 10.3390/polym18172119 (PMC13567974; doi:10.3390/polym18172119)
Supplement: Supplementary file 1 [file polymers-18-02119-s001.zip › polymers-4446865-supplementary.pdf]

SUPPLEMENTARY INFORMATION

Recycling Poly(butylene succinate)/Brewer's Spent Grain Pot Prototypes

Annamaria Visco*, Gianmarco Sindona*, Cristina Scolaro and Salim Brahimi

Department of Engineering, University of Messina, C.da Di Dio, 98166 Messina, Italy; cscolaro@unime.it (C.S.); salim.brahimi@unime.it (S.B.)

*Correspondence: avisco@unime.it (A.V.); gianmarco.sindona@unime.it (G.S.)

Tables and Figures Index

Figure S1. Comparison of FTIR Spectra of PBSv and PBSr (a), of PBSv, BSG, and MIXr (b), of PBSv, PBSr, MIXr, PBSv-MIXr10, PBSv-MIXr40, PBSv-PBSr10, and PBSv-PBSr40 (c)............................2

Table S1. Mechanical parameters of all materials, pure and blended, processed by melt mixing: Young modulus (E), tensile strength at yielding (σy) and at break (σr), elongation at yielding (εy) and at break (εr), work at break (W)...3

Table S2. TGA parameters of PBSv, PBSr, MIXr, and selected blends containing 10 wt.% and 40 wt.% of PBSr or MIXr...4

Table S3. Color parameters of PBSv-PBSr and of PBSv-MIXr blends.......................................5

Table S4. Surface roughness (Ra) and percentage increase in roughness compared to virgin PBS (Δ) of PBSv, PBSr, MIXr and all the blends...7

Table S5. Surface roughness parameters Rq and Rz of the PBSv/MIXr and PBSv/PBSr blends...7

Table S6. Summary of the test statistic of the Shapiro–Wilk normality test; p, probability value, performed for the investigated tensile properties...11

Table S7. Statistical summary normality test Brown-Forsythe ANOVA test or Welch's ANOVA; p, probability value...13

Table S8. Complete two-way ANOVA results (Bonferroni's multiple comparisons test) 15

Table S9. Mean difference ANOVA results (Bonferroni's multiple comparisons test) 18

Table S10. Regression analysis of the mechanical properties..20

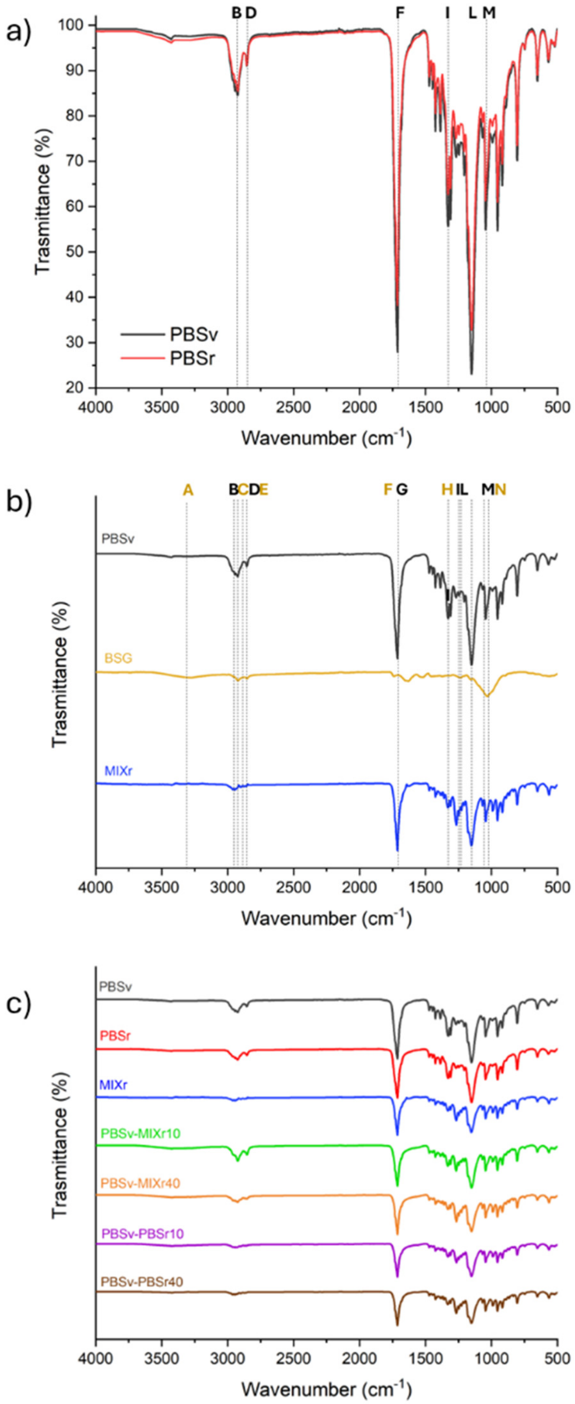

Figure S1. Comparison of FTIR Spectra of PBSv and PBSr (a), of PBSv, BSG, and MIXr (b), of PBSv, PBSr, MIXr, PBSv-MIXr10, PBSv-MIXr40, PBSv-PBSr10, and PBSv-PBSr40 (c).

Table S1. Mechanical parameters of all materials, pure and blended, processed by melt mixing: Young modulus (E), tensile strength at yielding (σ_y) and at break (σ_r), elongation at yielding (ε_y) and at break (ε_r), work at break (W).

	E (MPa)	σ_y (MPa)	ε_y (%)	σ_r (MPa)	ε_r (%)	W(J)
**PBSv**	394.38 ± 20.12	39.04 ±2.56	31.33 ±1.43	42.83 ±1.78	399.79 ± 27.54	5.90 ±0.03
**PBSv-PBSr2**	335.0 ± 12.01	35.27 ±1.90	24.6 ±3.89	43.48 ±3.22	512.36 ±22.39	6.71 ±0.04
**PBSv-PBSr5**	350.06 ±22.32	36.15 ±1.54	25.39 ±3.10	33.15 ±2.41	263.51 ±14.25	3.41 ±0.03
**PBSv-PBSr10**	379.64 ± 10.47	39.20 ±2.25	24.48 ±4.46	38.36 ±3.00	271.52 ±20.52	4.60 ±0.05
**PBSv-PBSr20**	366.91 ±14.57	-	-	33.91 ±2.20	23.98 ±2.27	0.24 ±0.02
**PBSv-PBSr30**	394.50 ±21.11	-	-	35.62 ±3.26	22.05 ±1.99	0.24 ±0.03
**PBSv-PBSr40**	381.94 ±14.98	-	-	36.03 ±2.50	21.75 ±3.30	0.23 ±0.02
**PBSr**	426.54 ±22.71	-	-	19.96 ±3.32	6.08 ±3.32	0.02 ±0.01
**PBSv-MIXr2**	359.30 ±18.31	-	-	28.61 ±2.95	32.72 ±4.49	0.35 ±0.02
**PBSv-MIXr5**	360.91 ±18.26	-	-	35.35 ±2.99	23.32 ±4.48	0.20 ±0.03
**PBSv-MIXr10**	371.03 ±9.86	-	-	33.40 ±2.42	20.64 ±5.39	0.19 ±0.04
**PBSv-MIXr20**	364.28 ±18.10	-	-	31.24 ±2.73	21.77 ±6.35	0.20 ±0.03
**PBSv-MIXr30**	382.58 ±11.95	-	-	26.11 ±2.83	17.75 ±5.65	0.13 ±0.01
**PBSv-MIXr40**	385.12 ±10.43	-	-	25.28 ±2.18	16.42 ±5.50	0.10 ±0.01
**MIXr**	507.52 ±12.97	-	-	12.12 ±1.66	4.40 ±2.01	0.01 ±0.01

Table S2. TGA parameters of PBSv, PBSr, MIXr, and selected blends containing 10 wt.% and 40 wt.% of PBSr or MIXr.

Sample	T_{onset}	1st Decomposition (BSG Phase)			2nd Decomposition (PBS Phase)			Residue at 700°C
	[°C]	T_{max1} [°C]	Δw_1 [%]	DTG Peak 1 [%/°C]	T_{max2} [°C]	Δw_2 [%]	DTG Peak 2 [%/°C]	[%]
**PBSv**	326±0.59	-	-	-	393± 2.06	92.38±0.13	2.21±0.005	2.93±0.20
**PBSv-PBSr10**	318±1.50	-	-	-	402±1.33	95.16±0.10	1.86±0.003	1.06±0.17
**PBSv-PBSr40**	319±0.90	-	-	-	402±1.64	95.51±0.25	1.97±0.005	0.28±0.12
**PBSr**	315±1.77	-	-	-	400±1.33	94.89±0.21	2.12±0.004	2.29±0.20
**PBSv-MIXr10**	273±0.85	297±1.50	1.08±0.39	0.04±0.36	401±1.06	95.83±0.26	2.28±0.004	0.54±0.10
**PBSv-MIXr40**	256±1.48	307±1.00	5.36±0.61	0.1±0.11	398±1.71	91.76±0.77	2.13±0.009	0
**MIXr**	239±1.01	290±2.47	11.63±0.83	0.18±0.07	396±1.26	79.54±0.52	1.7±0.007	5.23±0.56

Table S3 - Color parameters of PBSv-PBSr and of PBSv-MIXr blends.

Samples	L*	a*	b*	ΔE	Chroma	hue (◦)	B index	W index	Y index	Color
PBSv	72.55	-0.70	2.56	0.00	2.66	-74.70	0.31	72.42	5.05	
PBSv-PBSr2	81.36	-0.51	1.36	8.90	1.45	-69.46	0.31	81.31	2.38	
PBSv-PBSr5	79.96	-0.48	1.54	7.48	1.61	-72.82	0.31	79.90	2.74	
PBSv-PBSr10	79.83	-0.40	1.62	7.35	1.67	-76.02	0.31	79.76	2.90	
PBSv-PBSr20	80.52	-0.46	1.44	8.05	1.51	-72.34	0.31	80.46	2.56	
PBSv-PBSr30	80.50	-0.53	1.58	8.01	1.67	-71.29	0.31	80.43	2.80	
PBSv-PBSr40	77.13	-0.17	2.95	4.62	2.96	-86.66	0.31	76.94	5.47	
PBSr	77.62	-0.26	2.13	5.10	2.15	-83.11	0.31	77.52	3.93	
PBSv	72.55	-0.70	2.56	0.00	2.66	-74.70	0.31	72.42	5.05	
PBSv-MIXr2	68.43	1.19	10.72	9.33	10.78	83.68	0.31	66.64	22.37	
PBSv-MIXr5	64.07	3.74	15.67	16.23	16.11	76.56	0.32	60.63	34.94	
PBSv-MIXr10	54.13	13.20	20.42	29.18	24.31	57.11	0.35	48.08	53.88	
PBSv-MIXr20	48.69	9.21	20.52	31.46	22.49	65.83	0.34	43.98	60.20	
PBSv-MIXr30	44.73	9.79	18.08	33.54	20.56	61.55	0.34	41.03	57.72	
PBSv-MIXr40	41.85	10.24	18.35	36.22	21.01	60.85	0.35	38.17	62.64	
MIXr	38.40	8.55	13.83	37.13	16.26	58.27	0.34	36.29	51.45	

L* =lightness
a*, b* = coordinates which define the chromatic components of the color
Chroma = intensity or saturation of the color
hue angle = angle value that determines the type of color in the wheel CieLAB system.
ΔE = color difference between two samples (with respect to the PBSv)
W index = whiteness Index or the degree to which a material appears white, relative to an ideal white reference [29].
B index = browning Index [29].
Y index = Yellow Index [29].
Color = obtained by the conversion of L*a*b* coordinates to RGB color system

Table S4 - Surface roughness (Ra) and percentage increase in roughness compared to virgin PBS (Δ) of PBSv, PBSr, MIXr and all the blends.

PBSv-MIXr				PBSv-PBSr			
Amount (%)	Sample	R_a (μm)	Δ (%)	Amount (%)	Sample	R_a (μm)	Δ (%)
0	PBSv	0.704 ± 0.003	0.00	0	PBSv	0.704 ± 0.003	0.00
2	PBSv-MIXr2	0.771 ± 0.032	9.56	2	PBSv-PBSr2	0.732 ± 0.009	3.98
5	PBSv-MIXr5	0.802 ± 0.059	13.92	5	PBSv-PBSr5	0.755 ± 0.041	7.24
10	PBSv-MIXr10	0.864 ± 0.005	22.68	10	PBSv-PBSr10	0.782 ± 0.039	11.03
20	PBSv-MIXr20	0.902 ± 0.040	28.08	20	PBSv-PBSr20	0.823 ± 0.035	16.90
30	PBSv-MIXr30	0.919 ± 0.028	30.54	30	PBSv-PBSr30	0.861 ± 0.025	22.25
40	PBSv-MIXr40	0.928 ± 0.018	31.87	40	PBSv-PBSr40	0.882 ± 0.019	25.24
100	MIXr	1.116 ± 0.054	58.57	100	PBSr	0.961 ± 0.035	36.51

Table S5 - Surface roughness parameters Rq and Rz of the PBSv/MIXr and PBSv/PBSr blends.

PBSv-MIXr				PBSv-PBSr		
Amount (%)	Sample	Rq (μm)	Rz (μm)	Sample	Rq (μm)	Rz (μm)
0	PBSv	0.914 ± 0.043	5.782 ± 1.110	PBSv	0.914 ± 0.043	5.871 ± 1.110
2	PBSv-MIXr2	1.056 ± 0.040	5.893 ±0.770	PBSv-PBSr2	0.913 ± 0.027	4.570 ± 0.515
5	PBSv-MIXr5	1.089 ± 0.078	6.963 ± 0.738	PBSv-PBSr5	0.992 ± 0.071	6.961 ± 1.426
10	PBSv-MIXr10	1.104 ± 0.048	6.189 ± 1.055	PBSv-PBSr10	1.050 ± 0.047	6.881 ± 0.693
20	PBSv-MIXr20	1.232 ± 0.053	7.539 ± 1.392	PBSv-PBSr20	1.070 ± 0.054	6.161 ± 0.090
30	PBSv-MIXr30	1.185 ± 0.031	7.979 ± 0.966	PBSv-PBSr30	1.064 ± 0.018	5.798 ± 0.459
40	PBSv-MIXr40	1.240 ± 0.070	7.507 ± 0.787	PBSv-PBSr40	1.114 ± 0.025	6.374 ± 0.858
100	MIXr	1.656 ± 0.007	10.323 ± 0.724	PBSr	1.183 ± 0.037	5.452 ± 0.194

The incorporation of MIXr led to a progressive increase in surface roughness. Ra increased from 0.704 ± 0.003 μm for neat PBSv to 1.116 ± 0.054 μm for neat MIXr, while Rq increased from 0.914 ± 0.043 to 1.656 ± 0.007 μm. Although minor fluctuations were observed at intermediate compositions, the overall trend indicates that increasing MIXr content promotes the development of a rougher surface. Similarly, Rz increased from 5.78 to 10.32 μm, suggesting that the maximum peak-to-valley height also becomes larger as the MIXr fraction increases.
The average roughness parameters (Ra and Rq) exhibited a tendency to increase with increasing PBSr content. Ra increased from 0.704 ± 0.003 μm for PBSv to 0.961 ± 0.035 μm for PBSr, while Rq increased from 0.914 ± 0.043 μm to 1.183 ± 0.037 μm. In contrast, Rz fluctuated between 4.57 and 6.96 μm without a clear monotonic trend, suggesting that peak-to-valley heights are more affected by local surface heterogeneity than by blend composition.

Comprehensive Statistical Analysis of the mechanical tensile properties

The experimental design adopted in this study consist of predefined recycled-content formulations (2, 5, 10, 20, 30 and 40 wt%), independently manufactured and mechanically characterized. Recycled content was not sampled as a continuous random variable but implemented as fixed experimental treatment levels within a factorial design.
Specifically, the factorial ANOVA evaluates:

- the main effect of material formulation (PBSv–PBSr vs. PBSv–MIXr);
- the main effect of the recycled-content level;
- the interaction between material formulation and recycled-content level.

This interaction constitutes one of the principal objectives of the study and cannot be evaluated using a simple regression model.
Before inferential analyses, the assumptions of parametric statistics were systematically verified.
Residual normality was assessed using four complementary tests:

- Shapiro–Wilk;
- Anderson–Darling;
- D'Agostino–Pearson omnibus;
- Kolmogorov–Smirnov.

Homogeneity of variances was evaluated using the Brown–Forsythe test.
The analyses showed that the residuals satisfied the normality assumption for all investigated mechanical properties. Variance homogeneity was verified for the elastic and yield-related properties, whereas fracture-related properties—particularly strain at break (εr)—showed heteroscedasticity, which is consistent with the increased variability typically associated with tensile fracture.
To verify the robustness of the statistical conclusions, Welch's ANOVA was additionally performed for εr, confirming that the statistical significance remained unchanged despite unequal variances.
The revised manuscript now reports the complete results of the factorial ANOVA (Table S6-S10):

- F statistics;
- degrees of freedom;
- exact p-values;
- effect sizes (where available);
- interpretation of the interaction between material formulation and recycled-content level.

The analyses demonstrated significant effects of both factors and, for the fracture-related properties, significant material × recycled-content interactions, indicating that PBSv–PBSr and . PBSv–MIXr do not follow identical degradation pathways during recycling.
Bonferroni-adjusted post-hoc comparisons have now been fully reported and discussed.
These analyses identify the recycled-content levels at which PBSv–PBSr and . PBSv–MIXr differ significantly and complement the interpretation of the interaction observed in the two-way ANOVA.
For each mechanical property, the following information has been added:

- regression model;

- regression equation;
- coefficient of determination (R^2);
- goodness-of-fit statistics.

The regression analyses complement the factorial ANOVA by describing the continuous evolution of the mechanical properties as recycled content increases.

Importantly, the regression analyses confirmed the progressive deterioration of the investigated tensile properties. Young's modulus exhibited the lowest sensitivity to recycled content, whereas fracture-related properties—particularly strain at break (εr) and work to break (W)—showed the steepest degradation trends, consistent with the progressive transition from ductile to brittle mechanical behaviour discussed in the manuscript.

Residual diagnostics were examined during the regression analyses.

No influential observations requiring exclusion were identified, and all experimental data were therefore retained in the statistical analyses.

Overall, the statistical section has been extensively revised. The manuscript now combines factorial ANOVA, Bonferroni multiple comparisons, assumption testing and regression modelling, providing complementary statistical information.

The factorial ANOVA quantifies the effects of material formulation, recycled-content level and their interaction, whereas regression analysis describes the continuous evolution of each mechanical property with increasing recycled content.

Table S6. Summary of the test statistic of the Shapiro–Wilk normality test; *p*, probability value, performed for the investigated tensile properties

Code	E (MPa)	σr (MPa)	εr (%)	W(J)
**PBSv**	W= 0.9524<br>p= 0.7352	W=0.9184<br>p=0.4174	W=0.9541<br>p=0.7522	W=0.9248<br>p==0.4704
**PBSr**	W= 0.8717<br>p= 0.5341	W= 0.9295<br>p=0.5119	W=0.9049<br>p= 0.3197	W= 0.9267<br>p=0.4865
**MIX**	W= 0.9445<br>p= 0.6557	W= 0.9843<br>p=0.9809	W=0.8706<br>p=0.1528	W= 0.9214<br>p=0.4417
**PBSv-PBSr2**	W= 0.8284<br>p= 0.1566	W=0.8803<br>p=0.1895	W=0.8992<br>p=0.2844	W= 0.9193<br>p=0.4241
**PBSv-PBSr5**	W= 0.8537<br>p= 0.0572	W= 0.9381<br>p=0.5926	W=0.9204<br>p=0.4334	W= 0.9672<br>p=0.8750
**PBSv-PBSr10**	W= 0.9616<br>p= 0.1038	W= 0.9264<br>p=0.4842	W= 0.9567<br>p=0.7786	W= 0.9238<br>p=0.4619
**PBSv-PBSr20**	W= 0.9252<br>p= 0.8248	W= 0.9403<br>p=0.6141	W= 0.9680<br>p=0.8821	W= 0.9258<br>p=0.4786
**PBSv-PBSr30**	W= 0.9180<br>p= 0.4732	W= 0.8631<br>p=0.1290	W= 0.8786<br>p=0.1826	W= 0.9795<br>p=0.9604
**PBSv-PBSr40**	W= 0.9320<br>p= 0.4138	W= 0.9760<br>p=0.9403	W= 0.9398<br>p=0.6088	W= 0.9425<br>p=0.6360
**PBSv-MIX2**	W= 0.9484<br>p= 0.6952	W= 0.9339<br>p=0.5520	W=0.8820<br>p=0.1969	W= 0.8547<br>p=0.1063
**PBSv-MIX5**	W= 0.9198<br>p= 0.4284	W= 0.8974<br>p=0.2737	W=0.9590<br>p=0.8001	W= 0.9560<br>p=0.7712
**PBSv-MIX10**	W= 0.9062<br>p= 0.3281	W= 0.9442<br>p=0.6528	W= 0.9233<br>p=0.4574	W= 0.9040<br>p=0.3138
**PBSv-MIX20**	W= 0.9615<br>p= 0.8246	W= 0.9053<br>p=0.3223	W= 0.8932<br>p=0.2504	W= 0.9560<br>p=0.7712
**PBSv-MIX30**	W= 0.9495<br>p= 0.7062	W= 0.8961<br>p=0.2665	W= 0.9409<br>p=0.6195	W= 0.8822<br>p=0.1978
**PBSv-MIX40**	W= 0.9888<br>p= 0.9929	W= 0.9552<br>p=0.7635	W= 0.9389<br>p=0.5999	W= 0.8828<br>p=0.2004

W=test statistic of the Shapiro–Wilk normality test; p=probability value.

Discussion of Table S6 (Shapiro–Wilk normality test):

Before performing inferential statistical analyses, the distribution of the experimental data was evaluated using the Shapiro–Wilk normality test (Table S2). For all investigated tensile properties, including Young's modulus (E), stress at break (σr), strain at break (εr) and work at break (W), the null hypothesis of normality could not be rejected ($p > 0.05$ for all formulations), indicating that the experimental data were consistent with Gaussian distributions. This result was observed not only for the virgin materials (PBSv, PBSr and MIX), but also for all recycled formulations containing increasing amounts of PBSr or MIXr.

The absence of statistically significant deviations from normality demonstrates that the mechanical response of the investigated materials was sufficiently reproducible despite the intrinsic variability commonly associated with tensile testing of biodegradable polymers. Fracture-related properties such as elongation at break and work to break, which generally exhibit larger experimental scatter because of the stochastic nature of crack initiation and propagation, also satisfied the normality assumption across all investigated formulations. Consequently, the use of parametric statistical analyses was considered appropriate for all mechanical properties.

The Shapiro–Wilk results indicate that the observed differences among formulations originate from systematic changes induced by recycled content rather than from abnormal data distributions or isolated experimental outliers.

Table S7: Statistical summary normality test Brown-Forsythe ANOVA test or Welch's ANOVA; *p*, probability value.

	PBSv–PBSr					PBSv–MIXr				
**Mechanical property**	**Assumption tests**					**Assumption tests**				
**E (MPa)**	Brown-Forsythe ANOVA test	F*=20.27	DFn=7.000	DFd=45.47	P<0.0001	Brown-Forsythe ANOVA test	F*=79.68	DFn=7.000	DFd=45.61	P<0.0001
	Welch's ANOVA test	W=19.27	DFn=7.000	DFd=23.83	P<0.0001	Welch's ANOVA test	W=91.29	DFn=7.000	DFd=28.87	P<0.0001
**σr (Mpa)**	Brown-Forsythe ANOVA test	F*= 56.17	DFn=7.000	DFd= 49.35	P<0.0001	Brown-Forsythe ANOVA test	F*= 103.5	DFn=7.000	DFd=49.28	P<0.0001
	Welch's ANOVA test	W= 45.07	DFn=7.000	DFd= 23.89	P<0.0001	Welch's ANOVA test	W= 172.1	DFn=7.000	DFd=23.89	P<0.0001
**εr (%)**	Brown-Forsythe ANOVA test	F*=1327	DFn=7.000	DFd=24.55	P<0.0001	Brown-Forsythe ANOVA test	F*=1242	DFn=7.000	DFd=10.53	P<0.0001
	Welch's ANOVA test	W=1092	DFn=7.000	DFd=23.37	P<0.0001	Welch's ANOVA test	W=238.4	DFn=7.000	DFd=23.28	P<0.0001
**W (J)**	Brown-Forsythe ANOVA test	F*=60726	DFn=7.000	DFd=39.32	P<0.0001	Brown-Forsythe ANOVA test	F*=52496	DFn=7.000	DFd=36.98	P<0.0001
	Welch's ANOVA test	W=59240	DFn=7.000	DFd=23.35	P<0.0001	Welch's ANOVA test	W=26432	DFn=7.000	DFd=23.42	P<0.0001

F= statistic of Brown-Forsythe ANOVA test; DFn= numerator degrees of freedom; DFd=denominator degrees of freedom; W=test statistic of the Welch's ANOVA; p= probability value.*

Discussion on Table S7 (Brown–Forsythe and Welch ANOVA test):

Homogeneity of variances was subsequently evaluated using the Brown–Forsythe test (Table S7). For both PBSv–PBSr and PBSv–MIXr systems, statistically significant Brown–Forsythe statistics were obtained for all investigated tensile properties ($p < 0.0001$), indicating that the assumption of equal variances was not satisfied. The magnitude of the Brown–Forsythe statistics progressively increased from Young's modulus to the fracture-related parameters, demonstrating that variability became increasingly pronounced as the deformation mechanism shifted from elastic behaviour to fracture.

Because heteroscedasticity was detected, Welch's ANOVA was additionally performed for every mechanical parameter. In all cases, Welch's ANOVA also yielded highly significant results ($p < 0.0001$), confirming that the differences among the investigated recycled formulations remained statistically significant even after correction for unequal variances. Therefore, the conclusions derived from the subsequent analyses are robust and are not influenced by violations of the homogeneity assumption.

From a mechanical viewpoint, the progressive increase in variance observed for σr, εr and W reflects the increasing heterogeneity introduced by repeated thermo-mechanical recycling. While the elastic response (Young's modulus) remains comparatively stable, fracture-related properties become increasingly sensitive to local microstructural defects, chain scission and stress concentrations generated during recycling. These mechanisms result in greater specimen-to-specimen variability, particularly at high recycled contents where brittle fracture predominates.

Interestingly, both material systems exhibited the same statistical behaviour, although the Brown–Forsythe and Welch statistics were consistently larger for PBSv–MIXr than for PBSv–PBSr. This finding suggests that incorporating recycled MIX generates greater mechanical heterogeneity than recycling PBS alone, which is consistent with the lower ductility, reduced work to break and poorer interfacial stress transfer observed for the MIX-containing formulations.

Overall, the combination of significant Brown–Forsythe and Welch ANOVA results demonstrate that fracture-related mechanical properties are substantially more sensitive to recycling-induced microstructural heterogeneity than elastic stiffness.

Table 8. Complete two-way ANOVA results (Bonferroni's multiple comparisons test)

Mechanical Property	Source of variation	SS	DFn	DFd	MS	F	Exact p value	partial η^2
**E (MPa)**	Material	153.5	1	84	153.5	0.6183	0.4339	0.3374
	Recycled content	20868	5	84	4174	16.81	<0.0001	45.87
	Interaction (Material × Recycled content)	3613	5	84	722.7	2.910	0.0180	7.943
	Residual	20858	-	84	248.3	-	-	-
**σr (MPa)**	Material	1097	1	84	1097	145.2	<0.0001	37.23
	Recycled content	456.4	5	84	91.28	12.08	<0.0001	15.49
	Interaction (Material × Recycled content)	758.6	5	84	151.7	20.08	<0.0001	25.74
	Residual	634.8	-	84	7.557	-	-	-
**εr (%)**	Material	643598	1	84	643598	5864	<0.0001	28.56
	Recycled content	841499	5	84	168300	1533	<0.0001	37.34
	Interaction (Material × Recycled content)	759330	5	84	151866	1384	<0.0001	33.69
	Residual	9220	-	84	109.8	-	-	-
**W (J)**	Material	135.3	1	84	135.3	150882	<0.0001	30.61
	Recycled content	161.3	5	84	32.27	35986	<0.0001	36.50
	Interaction (Material × Recycled content)	145.3	5	84	29.07	32416	<0.0001	32.88
	Residual	0.07532	-	84	0.0008966	-	-	-

Summary of the two-way ANOVA performed to evaluate the effects of material type, recycled content, and their interaction on the mechanical properties of the investigated blends. SS= sum of squares; DFn= numerator degrees of freedom; DFd=denominator degrees of freedom; MS, mean square; F, F-statistic; p, exact probability value; partial η^2 = partial eta squared effect size, representing the proportion of variance explained by each factor after accounting for the residual error. Bonferroni's post-hoc test was used for pairwise multiple comparisons. Effect sizes were quantified using partial eta squared (partial η^2), allowing the relative contribution of each factor to be assessed independently of the residual variance. According to Cohen's guidelines, values of approximately 0.01, 0.06 and 0.14 were interpreted as small, medium and large effects, respectively.

Discussion of Table S8 (Bonferroni's multiple comparisons test):

Table S8 summarizes the two-way ANOVA performed to evaluate the independent and interactive effects of material type (PBSr or MIXr) and recycled content on the tensile mechanical properties of the investigated blends. In addition to statistical significance, the relative contribution of each factor was quantified using partial eta squared (partial η^2), providing a measure of effect size.

Young's modulus (E)

For Young's modulus, the main effect of material type was not statistically significant ($F(1,84) = 0.6183$, $p = 0.4339$, partial $\eta^2 = 0.34\%$), indicating that, on average, PBSv–PBSr and PBSv–MIXr exhibited comparable elastic stiffness. In contrast, recycled content exerted a highly significant influence ($F(5,84) = 16.81$, $p < 0.0001$, partial $\eta^2 = 45.87\%$), representing the principal source of variability in the elastic response. The significant interaction between material type and recycled content ($F(5,84) = 2.910$, $p = 0.0180$, partial $\eta^2 = 7.94\%$) indicates that the evolution of stiffness with increasing recycled fraction depends on the type of recycled material incorporated into the blend.

These findings suggest that the elastic response is primarily controlled by the amount of recycled material rather than by its origin. The slight increase in Young's modulus observed at high recycled contents is consistent with the reduced chain mobility caused by thermo-mechanical degradation, increased crystallinity resulting from shorter polymer chains, and the progressive reduction of the ductile amorphous phase.

Stress at break (σr)

Stress at break was significantly affected by all investigated factors. Material type showed a highly significant effect ($F(1,84) = 145.2$, $p < 0.0001$, partial $\eta^2 = 37.23\%$), while recycled content also contributed significantly ($F(5,84) = 12.08$, $p < 0.0001$, partial $\eta^2 = 15.49\%$). The interaction between the two factors was likewise highly significant ($F(5,84) = 20.08$, $p < 0.0001$, partial $\eta^2 = 25.74\%$).

The relatively large effect size associated with material type indicates that fracture strength is strongly dependent on the characteristics of the recycled polymer. Furthermore, the significant interaction demonstrates that the reduction in tensile strength with increasing recycled content differs between PBSr and MIXr formulations. This behaviour reflects differences in molecular degradation, phase morphology and interfacial stress transfer, which become increasingly important as recycled material replaces virgin PBS.

Strain at break (εr)

Among all investigated properties, strain at break exhibited the highest statistical sensitivity to recycling. Material type ($F = 5864$, $p < 0.0001$, partial $\eta^2 = 28.56\%$), recycled content ($F = 1533$, $p < 0.0001$, partial $\eta^2 = 37.34\%$) and their interaction ($F = 1384$, $p < 0.0001$, partial $\eta^2 = 33.69\%$) all produced extremely large and highly significant effects.

The comparable magnitude of the three partial η^2 values indicates that fracture ductility is simultaneously governed by the intrinsic characteristics of the recycled material, the recycled fraction and their combined interaction. Mechanically, this behaviour reflects the progressive transition from ductile to brittle fracture induced by repeated thermo-mechanical processing. Chain scission, molecular weight reduction and the formation of microstructural defects progressively limit plastic deformation before failure, leading to the dramatic decrease in elongation at break observed experimentally.

Work at break (W)

Work at break showed a statistical pattern closely resembling that of strain at break. Material type (F = 150882, p < 0.0001, partial η^2 = 30.61%), recycled content (F = 35986, p < 0.0001, partial η^2 = 36.50%) and their interaction (F = 32416, p < 0.0001, partial η^2 = 32.88%) all explained substantial proportions of the variance.

Since work at break represents the total mechanical energy absorbed prior to fracture, these results demonstrate that recycling progressively reduces the toughness of the material. The nearly balanced contribution of the three ANOVA terms indicates that energy absorption depends not only on the amount of recycled polymer but also on the intrinsic characteristics of the recycled phase and on the way both factors interact during deformation and crack propagation. The marked reduction in work at break is consistent with the simultaneous decrease in tensile strength and elongation at break, confirming that fracture-related properties are considerably more sensitive to recycling than elastic stiffness.

Overall, the two-way ANOVA demonstrates that the influence of recycling depends on the mechanical property considered. Young's modulus is predominantly controlled by recycled content, whereas fracture-related properties (σr, εr and W) are significantly affected by material type, recycled content and their interaction. The large partial η^2 values obtained for the fracture properties indicate that these variables account for a substantial proportion of the observed mechanical variability.

From a materials perspective, these results confirm that thermo-mechanical recycling does not substantially alter the elastic response of PBS-based blends but significantly modifies the mechanisms governing fracture. Increasing recycled content progressively reduces molecular integrity and promotes structural heterogeneity, resulting in lower fracture strength, reduced ductility and diminished energy absorption. The significant interaction effects further demonstrate that the evolution of mechanical performance cannot be described solely as a function of recycled content but also depends on the chemical composition and microstructural characteristics of the recycled material incorporated into the blend. This statistical evidence is fully consistent with the stress–strain curves and with the regression analyses, which show a gradual deterioration of fracture properties as the recycled fraction increases.

Table S9. Mean difference ANOVA results (Bonferroni's multiple comparisons test)

PBSv–PBSr vs. PBSv–MIXr	E (MPa) Mean 1	E (MPa) Mean 2	σr (MPa) Mean 1	σr (MPa) Mean 2	εr (%) Mean 1	εr (%) Mean 2	W (J) Mean 1	W (J) Mean 2
**2%**	335.0	359.3	43.48	28.61	512.4	32.72	6.714	0.3548
**5%**	350.1	360.9	33.15	35.35	263.5	23.32	3.415	0.2020
**10%**	379.6	371.0	38.36	33.40	271.5	20.64	4.588	0.1879
**20%**	366.9	364.3	33.91	31.24	23.98	21.77	0.2432	0.2020
**30%**	394.5	382.6	35.62	26.11	22.05	17.75	0.2393	0.1315
**40%**	381.9	385.1	36.03	25.28	21.75	16.42	0.2272	0.1032

Discussion of Table S9 (Comparison between PBSv–PBSr and PBSv–MIXr formulations):
Table S9 compares the mean mechanical properties of PBSv–PBSr and PBSv–MIXr blends at identical recycled contents, providing a direct evaluation of the influence of the recycled material on the tensile behaviour of the blends.
For Young's modulus (E), both material systems exhibited comparable stiffness throughout the investigated recycled-content range. At low recycled contents (2–10 wt.%), the differences between PBSv–PBSr and PBSv–MIXr remained limited, with values ranging from approximately 335–380 MPa and 359–371 MPa, respectively. At higher recycled contents (20–40 wt.%), both systems showed a slight increase in stiffness, reaching approximately 382–395 MPa. These observations are consistent with the two-way ANOVA, which identified recycled content as the dominant factor controlling the elastic response, whereas the main effect of material type was not statistically significant. The similar modulus values indicate that both recycled materials preserve the elastic rigidity of the virgin PBS matrix despite repeated thermo-mechanical processing.
In contrast, stress at break (σr) exhibited a more pronounced dependence on the recycled material. At 2 wt.% recycled content, PBSv–PBSr maintained a fracture strength of 43.48 MPa compared with only 28.61 MPa for PBSv–MIXr. Although the difference progressively decreased with increasing recycled content, PBSv–PBSr consistently retained higher fracture strength across the entire composition range. At 40 wt.% recycled material, fracture strength remained approximately 36 MPa for PBSv–PBSr but decreased to about 25 MPa for PBSv–MIXr. These results indicate that recycled PBS preserves load-bearing capability more effectively than recycled MIX, suggesting lower levels of molecular degradation and improved stress transfer during fracture.
The largest differences between the two material systems were observed for strain at break (εr). At 2 wt.% recycled content, PBSv–PBSr exhibited an elongation at break of approximately 512%, whereas PBSv–MIXr fractured at only about 33%. Increasing recycled content progressively reduced ductility in both systems; however, PBSv–PBSr maintained considerably higher

elongation values up to 10 wt.% recycled material. Beyond 20 wt.%, both formulations exhibited very limited ductility (approximately 16–24%), indicating a transition from ductile to predominantly brittle fracture. These observations agree with the large interaction effects identified by the two-way ANOVA and demonstrate that fracture ductility is strongly influenced by both recycled content and recycled material type.
A similar trend was observed for work at break (W), which integrates the energy absorbed during deformation until fracture. PBSv–PBSr exhibited substantially higher work-to-break values than PBSv–MIXr over the entire composition range. At 2 wt.% recycled content, PBSv–PBSr absorbed approximately 6.71 J, whereas PBSv–MIXr absorbed only 0.35 J, corresponding to an almost twenty-fold reduction in fracture energy. As recycled content increased, work at break progressively decreased in both systems, reaching approximately 0.23 J for PBSv–PBSr and 0.10 J for PBSv–MIXr at 40 wt.% recycled material. The parallel decrease in work at break and elongation at break confirms that fracture toughness is the mechanical property most strongly affected by recycling-induced degradation.
Overall, Table S9 demonstrates that, although both recycled materials produce comparable elastic stiffness, their influence on fracture behaviour differs substantially. PBSv–PBSr consistently exhibits higher fracture strength, greater elongation at break and higher energy absorption than PBSv–MIXr at the same recycled content. These findings indicate that the recycled PBS fraction retains a greater proportion of its original molecular integrity, whereas the recycled MIX material undergoes more extensive degradation and generates a more heterogeneous microstructure. Consequently, the mechanical performance of the blends is governed not only by the amount of recycled material incorporated but also by its composition and degradation state, supporting the significant interaction effects identified by the two-way ANOVA.

Table S10. Regression analysis of the mechanical properties.

Mechanical Property	Code	Best fit	Equation	R^2	F	DFn	DFd	Exact p	Slope significantly different from zero	pooled slope	pooled intercept
**E (MPa)**	**PBSv–PBSr**	Linear Regression	Y = 0.6442*X + 361.9	0.5627	7.719	1	6	0.0321	significant		
	**PBSv–MIXr**	Linear Regression	Y = 1.341*X + 355.9	0.8256	28.41	1	6	0.0018	significant		
	**Comparison of slopes**				4.148	1	12	0.0644	not significantly different	0.9926	
	**Comparison of intercepts**				1.037	1	13	0.3271	not significantly different		358.9
**σr (MPa)**	**PBSv–PBSr**	Linear Regression	Y = -0.1969*X + 40.51	0.7928	22.96	1	6	0.0030	significant	-0.2210	
	**PBSv–MIXr**	Linear Regression	Y = -0.2450*X + 35.71	0.8192	27.19	1	6	0.0020	significant		0.7246%
	**Comparison of slopes**				0.5925	1	12	0.4563	not significantly different		
	**Comparison of intercepts**				10.11	1	13	0.0072	very significant		
**εr (%)**	**PBSv–PBSr**	Non Linear fit_ One phase decay	Y = = 417 * EXP(-0.08565 * X)					0.8857	Not Significant		
	**PBSv–MIXr**	Non Linear fit_ One phase decay	Y = 399.8 * EXP(-1.239 * X					>0.9999	Not Significant		
**W (J)**	**PBSv–PBSr**	Non Linear fit_ One	Y = 5.98 * EXP(-0.1114 * X)					0.9286	Not Significant		

		phase decay									
	**PBSv–MIXr**	Non Linear fit_ One phase decay	Y = 5.895 * EXP(-1.399 * X)					>0.9999	Not Significant		

The table reports the regression equations, goodness-of-fit statistics (R^2), F-statistics, numerator (DFn) and denominator (DFd) degrees of freedom, probability values (p), and comparison of regression lines. E and σr, for both formulations, exhibited statistically significant positive linear trends, whereas neither the slopes nor the intercepts differed significantly between the two regression models.

Discussion on the regression analysis of the mechanical properties (Table S10)

Since recycled content represents a continuous quantitative variable, regression analyses were performed to investigate the relationship between recycled fraction and the tensile properties of the investigated blends. The regression analysis complements the two-way ANOVA by describing the degradation trend associated with increasing recycled content, whereas the ANOVA identifies statistically significant differences among the discrete experimental formulations.

Young's modulus (E)

Young's modulus exhibited a statistically significant positive linear relationship with recycled content for both material systems. For the PBSv–PBSr blends, the best-fitting model was Y = 0.6442X + 361.9 (R^2 = 0.5627; F = 7.719; p = 0.0321), whereas the PBSv–MIXr blends followed the equation Y = 1.341X + 355.9 (R^2 = 0.8256; F = 28.41; p = 0.0018). In both cases, the slopes were significantly different from zero, confirming a progressive increase in stiffness with increasing recycled content. However, comparison of the regression lines indicated that neither the slopes (F = 4.148, p = 0.0644) nor the intercepts (F = 1.037, p = 0.3271) differed significantly between the two formulations.

Mechanically, the increase in Young's modulus can be attributed to the reduced molecular mobility resulting from thermo-mechanical degradation. Chain scission generates shorter polymer chains that crystallize more readily, increasing the effective stiffness of the material despite the progressive deterioration of fracture properties. The absence of significant differences between the two regression lines indicates that the increase in stiffness follows a comparable trend irrespective of whether the recycled fraction consists of PBSr or MIXr.

Stress at break (σr)

Stress at break also followed a statistically significant linear trend. PBSv–PBSr blends were described by the regression equation Y = −0.1969X + 40.51 (R^2 = 0.7928; F = 22.96; p = 0.0030), while PBSv–MIXr blends followed Y = −0.2450X + 35.71 (R^2 = 0.8192; F = 27.19; p = 0.0020). Both negative slopes were significantly different from zero, demonstrating that fracture strength decreases progressively with increasing recycled content.

Comparison of the regression lines showed no significant difference between the slopes (F = 0.5925, p = 0.4563), indicating that both recycled systems lose fracture strength at a similar rate as recycled content increases. In contrast, the intercepts differed significantly (F = 10.11, p = 0.0072), indicating that PBSv–PBSr exhibits consistently higher fracture strength than PBSv–MIXr throughout the investigated composition range. This result confirms that the intrinsic characteristics of the recycled material influence the absolute mechanical performance, whereas the degradation rate with increasing recycled fraction remains comparable.

Strain at break (εr)

Unlike Young's modulus and stress at break, strain at break was best described by a one-phase exponential decay model rather than by a linear regression. PBSv–PBSr followed the equation Y = 417 × exp(−0.08565X) (R^2 = 0.8857), whereas PBSv–MIXr was described by Y = 399.8 × exp(−1.239X) with an almost perfect fit (R^2 > 0.9999).

The exponential behaviour indicates that ductility decreases rapidly even at low recycled contents before approaching an asymptotic minimum at higher recycled fractions. This trend reflects the progressive transition from ductile to brittle fracture caused by chain scission,

molecular weight reduction and the accumulation of structural defects during repeated thermo-mechanical processing. The considerably higher decay constant observed for PBSv–MIXr demonstrates a much faster loss of ductility than in PBSv–PBSr, highlighting the greater susceptibility of the MIX-containing formulations to recycling-induced embrittlement.

Work at break (W)

Work at break also followed a one-phase exponential decay, with PBSv–PBSr described by $Y = 5.98 \times \exp(-0.1114X)$ ($R^2 = 0.9286$) and PBSv–MIXr by $Y = 5.895 \times \exp(-1.399X)$ ($R^2 > 0.9999$).

Since work at break represents the total mechanical energy absorbed before fracture, the exponential decrease demonstrates that recycling rapidly reduces material toughness. The much steeper decay observed for PBSv–MIXr indicates that the presence of mixed recycled material accelerates the loss of energy absorption capability, most likely because of greater microstructural heterogeneity and less efficient stress transfer during deformation. Consequently, while both recycled systems become progressively more brittle as recycled content increases, the deterioration is substantially more severe for MIX-containing blends.

The regression analysis demonstrates that the influence of recycled content depends on the mechanical property considered. Elastic stiffness follows a moderate positive linear trend, indicating that recycling slightly increases rigidity. Conversely, fracture-related properties exhibit progressive degradation with increasing recycled content. Stress at break decreases linearly, whereas strain at break and work at break follow exponential decay functions, demonstrating that ductility and toughness are considerably more sensitive to recycling than stiffness. Together with the two-way ANOVA, these results show that recycled content determines the overall degradation trend, while material composition primarily influences the absolute mechanical performance. This combination of statistical approaches provides a comprehensive description of how thermo-mechanical recycling affects the tensile behaviour of PBS-based blends.
